# Synthesis of Photoresponsive Dual NIR Two-Photon Absorptive [60]Fullerene Triads and Tetrads

**DOI:** 10.3390/molecules18089603

**Published:** 2013-08-12

**Authors:** Seaho Jeon, Min Wang, Loon-Seng Tan, Thomas Cooper, Michael R. Hamblin, Long Y. Chiang

**Affiliations:** 1 Department of Chemistry, Institute of Nanoscience and Engineering Technology, University of Massachusetts, Lowell, MA 01854, USA; 2 AFRL/RXAS, Functional Materials Division, Air Force Research Laboratory, Wright-Patterson Air Force Base, Dayton, OH 45433, USA; 3 Wellman Center for Photomedicine, Massachusetts General Hospital, Department of Dermatology, Harvard Medical School, Boston, MA 02114, USA; 4 Harvard MIT Division of Health Science and Technology, Cambridge, MA 02139, USA

**Keywords:** C_60_-(antenna)_x_ nanostructures, ultrafast intramolecular energy-transfer, NIR two-photon absorption, 2*γ*-photodynamic therapeutic agent, photosensitizer

## Abstract

Broadband nonlinear optical (NLO) organic nanostructures exhibiting both ultrafast photoresponse and a large cross-section of two-photon absorption throughout a wide NIR spectrum may make them suitable for use as nonlinear biophotonic materials. We report here the synthesis and characterization of two C_60_-(antenna)_x_ analogous compounds as branched triad C_60_(>DPAF-C_18_)(>CPAF-C_2M_) and tetrad C_60_(>DPAF-C_18_)(>CPAF-C_2M_)_2_ nanostructures. These compounds showed approximately equal extinction coefficients of optical absorption over 400–550 nm that corresponds to near-IR two-photon based excitation wavelengths at 780–1,100 nm. Accordingly, they may be utilized as potential precursor candidates to the active-core structures of photosensitizing nanodrugs for 2*γ*-PDT in the biological optical window of 800–1,050 nm.

## 1. Introduction

Fullerenes are nanocarbon cages with all sp^2^ carbons interlinked in a structure of hollow sphere. Highly strained curving regions of the cage surface consist of chemically reactive six fulvalenyl bridging olefins that can be utilized for making nucleophilic addition reactions. Chemical modification of C_60_ on only a limit number of functionalization sites may not lead to much alternation of the cage’s photophysical properties. Conversely, nucleophilic addition of one or two light-harvesting antenna chromophores will largely enhance the cage’s ability to respond and perform various photoinduced electronic and energy-related events by acting as an electron-acceptor [1,2]. The development of broadband nonlinear optical (NLO) organic nanostructures exhibiting both ultrafast photo-response and high efficiency in two-photon absorption throughout a wide NIR spectrum to variable laser pulses with duration ranging from fs to ns remains as the focus of nonlinear biophotonic materials. The goal requires the design of sophisticated, hydrophilic and biocompatible multifunctional NLO materials for two-photon absorption (2PA) based photodynamic therapy (2*γ*-PDT) [3,4,5,6,7] against pathogens and cancer to minimize the damage to surrounding normal tissue. Photoresponsive complex fullerene derivatives [8,9,10,11,12,13,14,15] and a number of organic chromophores [16,17,18,19] have been found to exhibit enhanced nonlinear photonic behavior. The control of photodynamic effect is precise due to the fact that 2*γ*-PDT can only be practiced at the focal area of the laser beam that prevents side-effects arising from the undesirable photokilling of normal cells. 

**Scheme 1:**
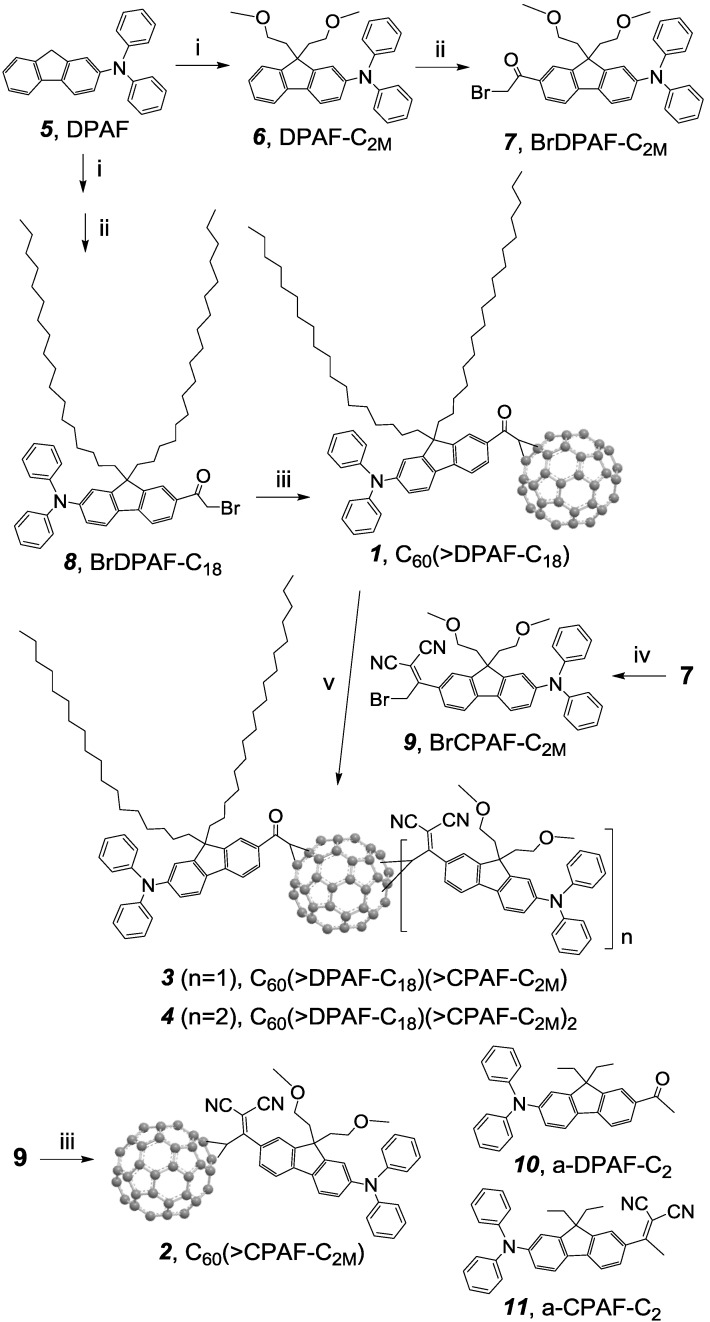
Synthesis of **3** and **4**.

The most abundant [60]fullerene is more readily available commercially in up to kilogram quantities than a number of higher fullerenes. However, its visible absorption extinction coefficient is rather low. This limitation can be overcome by attaching highly fluorescent chromophores as light-harvesting antenna units, such as porphyrin [20,21] or dialkyldiphenylaminofluorene (DPAF-C_n_), to enhance visible absorption of the resulting conjugates and, in the latter cases, 2PA cross-sections in the NIR wavelengths [10,13,14]. The absorbed photoenergy by the donor antenna was able to undergo efficient intramolecular transfer to the fullerene acceptor moiety, leading to the generation of excited triplet cage state ^3^(C_60_>)* after the intersystem crossing from its excited singlet state ^1^(C_60_>)*. Triplet energy transfer from ^3^(C_60_>)* to molecular oxygen produces singlet oxygen (^1^O_2_) that gives the cytotoxic effect to the cells in the Type-II photochemistry [22,23]. In this paper, we report the synthesis and spectroscopic characterization of photoresponsive dual NIR two-photon absorptive [60]fullerene triads and tetrads using the extended synthetic method for the preparation of their corresponding monoadduct analogous C_60_(>DPAF-C_18_) **1** and C_60_(>CPAF-C_2M_) **2**, as shown in Scheme 1. These triads and tetrads are capable of undergoing 2PA-based photoexcitation process at either 780 or 980 nm making them potential precursor candidates to the active-core structures of nanodrugs for 2*γ*-PDT.

## 2. Results and Discussion

Structural design of hybrid [60]fullerene triads and tetrads was based on both linear and nonlinear optical characteristics of 9,9-dioctadecyl-2-diphenylaminofluorenyl-61-carbonylmethano[60]fullerene (**1**), C_60_(>DPAF-C_18_) [24], and 9,9-di(2-methoxyethyl)-2-diphenylaminofluorenyl-61-(1,1-dicyano-ethylenyl)methano[60]fullerene (**2**), C_60_(>CPAF-C_2M_) [25], to construct an unique nanostructure system with a shared C_60_ cage. Specifically, covalent attachment of an antenna donor chromophore to a C_60_ molecule (electron-acceptor) was accomplished via a periconjugation linkage with a physical separation distance of only <3.5 Ǻ between the donor and acceptor moieties. This led to the realization of ultrafast intramolecular energy- and/or electron-transfer from photoexcited antenna moiety to C_60_ in <130–150 fs [14] that made this type of C_60_-antenna conjugates, C_60_(>DPAF-C_n_)_x_, capable of exhibiting photoresponse in a nearly instantaneous time scale to protect against high-intensity radiation. By increasing the number of attached antennae to four per C_60_ cage giving *starburst* pentad nanostructures, highly enhanced fs 2PA cross-section values were observed in a concentration-dependent manner [26]. Upon the chemical alteration of the keto group of C_60_(>DPAF-C_n_) bridging between C_60_ and the antenna moiety to a highly electron-withdrawing 1,1-dicyanoethylenyl (DCE) group, it was possible to extend the *π*-conjugation in the resulting C_60_(>CPAF-C_n_) analogous chromophore molecules to a close contact with the cage current. This led to a large bathochromic shift of the linear optical absorption of C_60_(>CPAF-C_2_) moving from 410 nm (*λ*_max_) of the parent keto-compound to 503 nm with the shoulder band being extended beyond 550 nm in the UV-vis spectrum. The shift considerably increased its light-harvesting ability in visible wavelengths and caused a nearly 6-fold higher in the production quantum yield of singlet oxygen (^1^O_2_) from C_60_(>CPAF-C_2M_) as compared with that of C_60_(>DPAF-C_2M_). The mechanism of ^1^O_2_ production was originated from the intermolecular triplet-energy transfer from the ^3^(C_60_>)* cage moiety to ^3^O_2_. A large increase in the production of reactive oxygen species (ROS) by excited C_60_(>CPAF-C_2M_) explained its effective photokilling of HeLa cells* in vitro*, via 1*γ*-PDT [25]. The observation demonstrated the intramolecular/intramolecular interaction between the excited CPAF-C_n_ donor antenna moiety and the acceptor C_60_ cage that was also confirmed by transient absorption spectroscopic measurements using ns laser pulses at 480–500 nm [27]. The behavior resembles that of DPAF-C_n_ antenna with transient photoexcitation at 380–410 nm reported previously [28]. By extending the same intramolecular photophysical properties to 2PA-based excitation applications, these C_60_-(antenna)_x_ analogous nanostructures may be utilized as potential photosensitizers for 2*γ*-PDT at either 800 nm (with DPAF antenna) or 1,000 nm (with CPAF antenna) that is well-suited to the biological optical window of 800–1,100 nm. 

Accordingly, selective attachment of these two antenna moiety types DPAF-C_n_ and CPAF-C_n_ in combination as hybrid chromophore addends to a single C_60_ cage should result in the formation of new methano[60]fullerene triads, C_60_(>DPAF-C_18_)(>CPAF-C_2M_) **3**, and tetrads, C_60_(>DPAF-C_18_)(>CPAF-C_2M_)_2_ **4**, as shown in Scheme 1. The core chromophore moiety of **3** and **4** will then be capable of performing dual-band 2*γ*-PDT-based photoinduced biocidal effects with enhanced penetration depth at 800–1,100 nm. Synthetically, preparation of **3** and **4** was accomplished by the synthesis of a structurally well-defined monoadduct **1**, followed by the attachment of one or two CPAF-C_2M_ antenna in sequence. A key intermediate precursor, 7-*α*-bromoacetyl-9,9-dioctadecyl-2-diphenylaminofluorene (BrDPAF-C_18_, **8**) was prepared by a three-step reaction involving first palladium catalyzed diphenylamination of commercially available 2-bromofluorene at the C2 position of the fluorene ring to afford DPAF **5** (Scheme 1). It was followed by dialkylation at the C9 carbon position of **5** using 1-bromooctadecane as the reagent in the presence of potassium *t*-butoxide, as a base, in THF at 0–25 °C to give the corresponding 9,9-dioctadecyl-2-diphenylaminofluorene (DPAF-C_18_) in 97% yield. Friedel-Crafts acylation of DPAF-C_18_ with *α*-bromoacetyl bromide and AlCl_3_ in CH_2_Cl-CH_2_Cl at 0 °C for a period of 4.0 h afforded the compound **8** in a yield of 96%. Addition reaction of **8** to C_60_ was carried out in the presence of 1,8-diazabicyclo[5.4.0]undec-7-ene (DBU, 1.0 eq.) at ambient temperature for 4.0 h to result in C_60_(>DPAF-C_18_) **1** in 65% yield (based on recovered residual C_60_) after column chromatographic purification.

*Reagents and conditions*: (**i**) 2-MeOCH_2_CH_2_–OMs (for **6**), 1-C_18_H_37_Br (for **8**), *t*-BuOK in THF, 0 °C–r.t., 4 h; (**ii**) *α*-bromoacetyl bromide, AlCl_3_, ClCH_2_CH_2_Cl, 0 °C, 4 h; (**iii**) DBU, toluene, r.t., 4 h; (**iv**) TiCl_4_, pyridine, CH_2_(CN)_2_, CHCl_3_, r.t., 5.0 min; (**v**) DBU, toluene, r.t., 4 h.

A similar reaction sequence was applied for the synthesis of the compound **2** by replacing two octadecyl groups with 2-methoxyethyl groups. Thus, 2-methoxyethyl methanesulfonate was used as a leaving group for dialkylation of DPAF **5** followed by Friedel-Crafts acylation with *α*-bromoacetyl bromide and AlCl_3_ to yield 7-*α*-bromoacetyl-9,9-di(2-methoxy)ethyl-2-diphenylaminofluorene (BrDPAF-C_2M_, **7**), Subsequent conversion of the keto group of **7** to the corresponding 1,1-dicyano-ethylenyl (DCE) group was carried out by the reaction using malononitrile as a reagent, pyridine as a base, and titanium tetrachloride as a deoxygenation agent in dry chloroform at ambient temperature for a short period of 5.0 min. The reaction resulted in the corresponding diphenylaminofluorene BrCPAF-C_2M_ **9** in a yield of 89% after chromatographic purification (PTLC, SiO_2_, CHCl_3_ as the eluent). Attachment of a CPAF-C_2M_ antenna arm to a C_60_ cage was carried out by identical reaction conditions as those for **1** with DBU (1.0 eq.) at room temperature for 4.0 h to afford 7-(1,2-dihydro-1,2-methano[60]fullerene-61-{1,1-dicyanoethylenyl})-9,9-di(2-methoxyethyl)-2-diphenylaminofluorene C_60_(>CPAF-C_2M_), **2**) as orange red solids in 53% yield (based on recovered C_60_). The bulkiness of DPAF-C_18_ and CPAF-C_2M_ in size can prevent these two types of antenna moieties form locating in close vicinity to each other at the cage surface. By considering the regio-location of reactive bicyclopentadienyl olefin bonds on the fullerene surface, when the first antenna is bound at the north-pole location, the second antenna arm is most likely to be pushed away to the equator area of the C_60_ sphere. Therefore, only a very limited number of multiadduct regioisomers per C_60_ are likely to form. Indeed, by controlling the reaction kinetic rate with two molar equivalents of CPAF-C_2M_ applied in the reaction with **1** in the presence of DBU (2.0 eq.), only two clear PTLC (SiO_2_, toluene–ethyl acetate/9:1 as the eluent) bands in the product mixtures were observed in addition to the starting **1** (~15%). The first less polar product band at *R*_f_ = 0.5 was found to be the bisadduct C_60_(>DPAF-C_18_)(>CPAF-C_2M_) **3** isolated as orange-brown solids in 28% yield. The second more polar product band at *R*_f_ = 0.4 (toluene–ethyl acetate/4:1 as the eluent) was determined to be the trisadduct C_60_(>DPAF-C_18_)(>CPAF-C_2M_)_2_ **4** isolated as red-brown solids in 40% yield.

**Figure 1:**
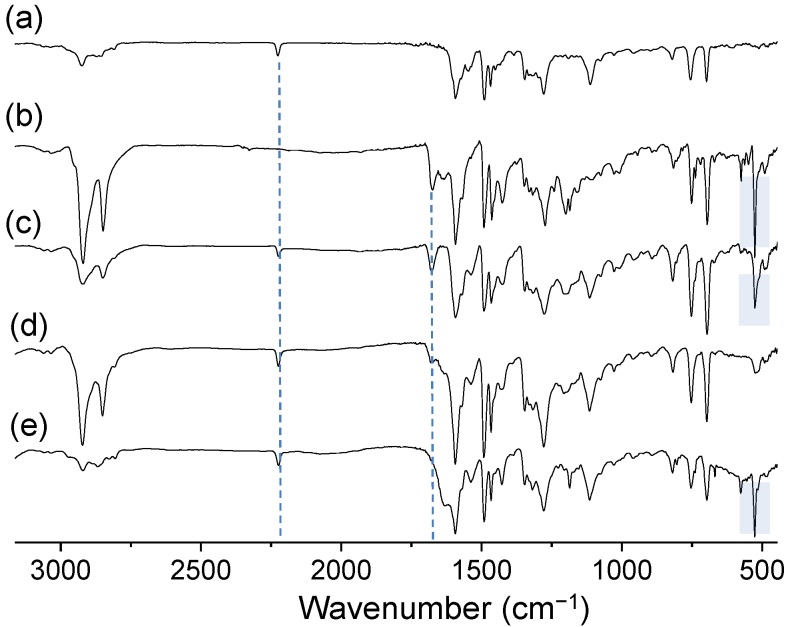
Infrared spectra of (**a**) BrCPAF-C_2M_ **9**, (**b**) C_60_(>DPAF-C_18_) **1**, (**c**) C_60_(>DPAF-C_2M_)(>CPAF-C_2M_) **3**, (**d**) C_60_(>DPAF-C_18_)(>CPAF-C_2M_)_2_ **4**, and (**e**) C_60_(>CPAF-C_2M_) **2**.

**Figure 2:**
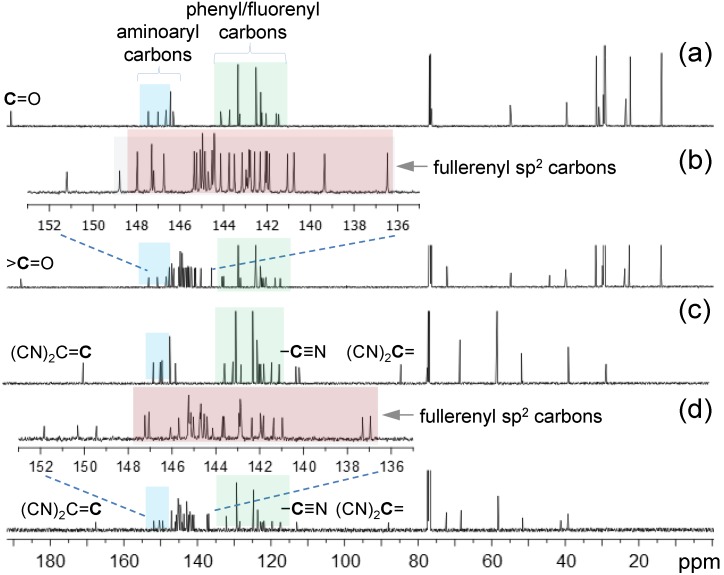
^13^C-NMR spectra of (**a**) BrDPAF-C_18_ **8**, (**b**) C_60_(>DPAF-C_18_) **1**, (**c**) BrCPAF-C_2M_ **9**, and (**d**) C_60_(>CPAF-C_2M_) **2** with three regions of carbon peaks marked by blue, green, and brown.

Spectroscopic characterization of **1** and **2** was performed mainly by: (i) the clear detection of a group of molecular mass ion peaks with the maximum peak intensity centered at *m/z* 1,600 (MH^+^ of **1**) and 1,258 (MH^+^ of **2**) (Supporting Information) using positive ion matrix-assisted laser desorption ionization (MALDI–TOF) mass spectroscopy and (ii) analyses of ^13^C-NMR spectra. The former spectra were also accompanied with two groups of fragmented mass ion peaks at *m/z* 720 and 734/735 corresponding to the mass units of C_60_ and C_60_>, respectively, indicating high stability of the fullerene cage under MALDI-MS conditions. In addition to the IR spectral analysis (Figure 1) of the carbonyl stretching vibration band at 1,674 cm^−1^ for **1** and the cyano (-C≡N) stretching band centered 2,224 cm^−1^ for **2**, chemical shifts of a keto carbonyl carbon peak at *δ* 188.33 and three carbons, **-C**=C(CN)_2_, –**C**≡N, and =**C**(CN)_2_, in 1,1-dicyanoethylenyl (DCE) moiety of **2** at δ 167.64, 112.99, and 88.07, respectively, in their ^13^C-NMR spectra [Figure 2(b) and (d)], clearly consistent with both structures. Chemical shift of the former carbonyl carbon peak agrees well with that of BrDPAF-C_18_ **8** at *δ* 190.99 [Figure 2(a)]. The *δ* values of the latter three DCE carbons were also found to match well with those of BrCPAF-C_2M_ **9** [Figure 2(c)] at *δ* 170.85, 112.98, (–**C**≡N), 112.11 (–**C**≡N), and 84.48, respectively. In the same spectra, the peaks at *δ* 40.14/41.22 and 72.48/72.30 were assigned to the cyclopropanyl or methanofullerene carbon C_61_ (C_60_>) and fullerenyl sp^3^ carbons of **1**/**2**, respectively. The rest of aromatic carbon peaks were separated from each other into three different groups with assigned chemical shifts of (i) three aminoaryl carbons of **1**/**2** at *δ* (153.55, 151.20, 148.77)/(151.83, 150.31, 149.45) in close resemblance to those of **8** and **9**, respectively, (ii) phenyl and fluorenyl carbons at *δ* 115–135, and (iii) fullerenyl sp^2^ carbons located at *δ* 136–148, as shown in Figure 2. A total of 30 fullerenyl carbon (28 × 2C and 2 × 1C) signals, some with similar or slightly shifted *δ*, were accounted for 58 sp^2^ fullerenyl carbons that fits well with a *C*_2_ molecular symmetry of the compounds **1** and **2**. 

**Figure 3:**
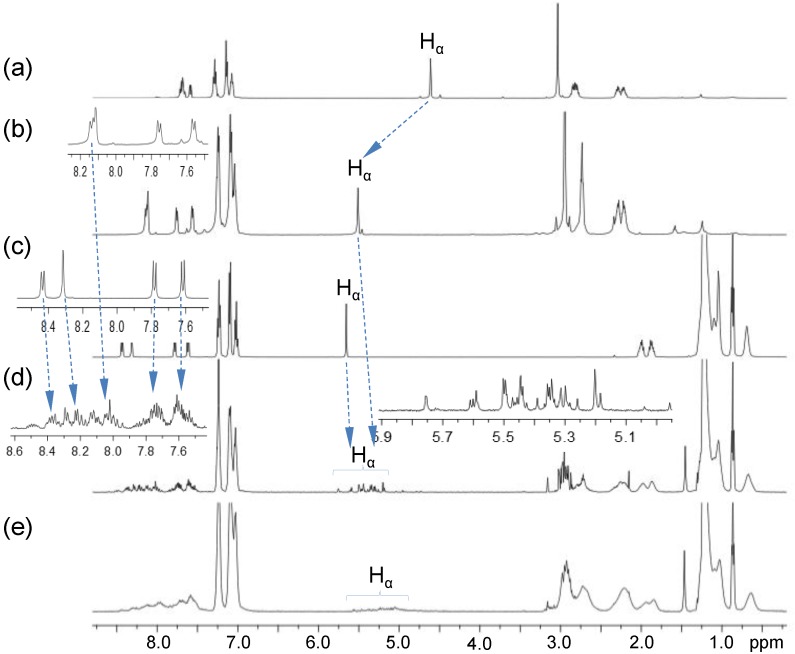
^1^H-NMR spectra (CDCl_3_) of (**a**) BrCPAF-C_2M_ **9**, (**b**) C_60_(>CPAF-C_2M_) **2**, (**c**) C_60_(>DPAF-C_18_) **1**, (**d**) C_60_(>DPAF-C_18_)(>CPAF-C_2M_) **3**, and (**e**) C_60_(>DPAF-C_18_)(>CPAF-C_2M_)_2_ **4**.

With well-characterized structures of **1** and **2**, we were able to utilize their ^1^H-NMR spectra for the correlation and identification of hybrid [60]fullerene triads **3** and tetrads **4**. Upon the attachment of one CPAF-C_2M_ antenna arm to **1**, a new cyano stretching band centered at 2,223 cm^−1^ in addition to the carbonyl stretching band at 1678 cm^−1^ were detected as expected. Intensity of characteristic half-fullerene cage absorption band at ~526 cm^−1^ was found to decrease significantly going from that of **1**, **3**, to **4** (Figure 1) indicating the increasing percentage of regioisomers having at least one CPAF-C_2M_ addend located at more than 90° away the DPAF-C_18_ arm (or the other side of the cage surface). Large difference of ^1^H chemical shifts among alkyl groups of DPAF-C_18_ (methyl and the most of methylene proton peaks at *δ* 0.69–1.29) and CPAF-C_2M_ (singlet terminal methoxy C**H**_3_-O– proton peak at *δ* 2.95 and triplet methylenoxy –C**H**_2_-O– proton peaks centered at *δ* 2.73) allowed us to measure a clear proton integration count to verify the structure of **3** and **4** as a bisadduct and trisadduct, respectively, as shown in Figure 3. A more branched structure of **4** was also evident by the detection of a higher aromatic proton integration ratio in the region of *δ* 7.5–7.8 and 8.10–8.15 [Figure 3(b) and (e)] of CPAF moieties. The most distinguishable proton peaks at *δ* 5.5–5.7 in these spectra were assigned for *α*-protons each bound on the cyclopropanyl carbon located between either the keto (for DPAF) or DCE (for CPAF) group and the C_60_ cage. Owing to the fullerenyl ring current, a large down-field shift of the *δ* value was observed at *δ* 5.66 (for the keto *α*-H) and 5.51 (for the DCE *α*-H) from that of the fluorenyl *α*-bromoketo *α*-H at *δ* 4.61 [Figure 3(a–c)] or *δ* 2.6 for the fluorenyl keto *α*-H (without *α*-attachment of a bromine atom, a large shift of ~3.0 ppm). It also caused a down-field *δ* shift of 0.44–0.48 ppm for fluorenyl protons located at the vicinity of C_60_> moiety that clearly revealed strong electronic interactions between DPAF-C_18_/CPAF-C_2M_ antenna moieties and the fullerene cage. 

**Figure 4:**
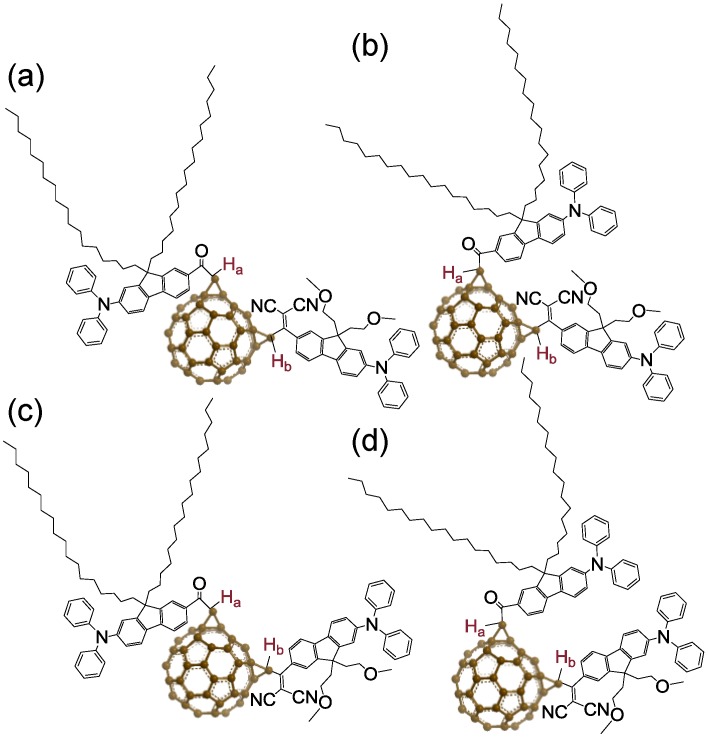
Four possible structural conformers for each regioisomer of C_60_(>DPAF-C_18_)(>CPAF-C_2M_) **3**.

**Figure 5:**
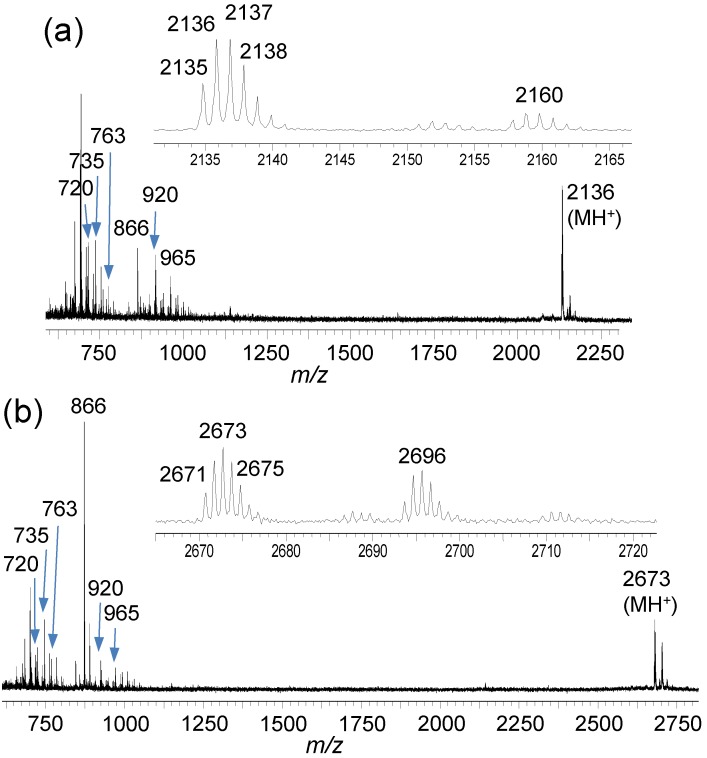
MALDI mass spectra of (**a**) C_60_(>DPAF-C_18_)(>CPAF-C_2M_) **3** and (**b**) C_60_(>DPAF-C_18_)(>CPAF-C_2M_)_2_ **4**.

A number of *α*-H peaks were observed in the ^1^H-NMR spectrum of **3** [the inset of Figure 3(d)]. By taking the consideration of four possible different orientational configurations for each regioisomer, as examples shown in Figure 4, one regioisomeric molecule may display four keto *α*-H_a_ peaks (from the DPAF-C_18_ moiety) and four DCE *α*-H_b_ peaks (from the CPAF-C_2M_ moiety) in the region of *δ* 5.0–5.75. Therefore, detected *α*-H_a_ peaks each in different intensities can be separately grouped into and accounted for two major regioisomer products and one minor regioisomer product. High similarity of molecular polarity among these regioisomers prohibited us to separate them chromatographically. However, we were able to confirm the identical composition mass of these regioisomers by detecting an group of sharp molecular mass ions with the maximum mass at *m/z* 2,136 (MH^+^), as shown in Figure 5(a). It was accompanied by a relatively simple MALDI-TOF mass spectrum showing fully fragmented mass ions at *m/z* 763, 735, and 720 corresponding clearly to the mass of C_60_[>(C=O)-H]H^+^, C_60_>H^+^, C_60_^+^, respectively, that was consistent well with the molecular structure of triad C_60_(>DPAF-C_18_)(>CPAF-C_2M_) **3**. In the case of tetrad C_60_(>DPAF-C_18_)(>CPAF-C_2M_)_2_ **4**, a group of sharp molecular mass ions with the maximum mass at *m/z* 2,673 (MH^+^) and similar fragmented mass ions to those of **3** in the low mass region of *m/z* 720–1,000 were detected [Figure 5(b)]. These MS data revealed high stability of aromatic diphenylaminofluorene moiety under measurement conditions. Additional high mass groups of peaks with the peak maximum at *m/z* 2160 of Figure 5(a) and *m/z* 2,696 of Figure 5(b) are satellite peaks with an increase of 2C (*m/z* 24) mass from those of molecular ion mass peaks, as common phenomena for fullerenyl nanocarbon materials, especially, under the high laser power conditions used for the collection of high mass ions. The fragmentation pattern fits well with the bond cleavage occurring mostly at the cyclopropanyl carbon bonds bridging the C_60_ cage and DPAF-C_18_/CPAF-C_2M_ antenna moiety. The overall spectra provided strong evidence for the mass composition of **3** and **4**. 

**Figure 6:**
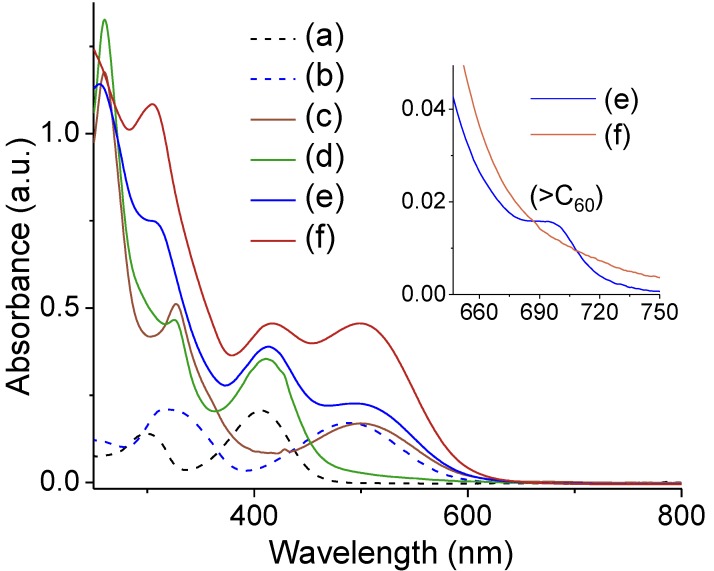
UV-vis spectra of (**a**) BrDPAF-C_18_ **8**, (**b**) BrCPAF-C_2M_ **9**, (**c**) C_60_(>CPAF-C_2M_) **2**, (**d**) C_60_(>DPAF-C_18_) **1**, (**e**) C_60_(>DPAF-C_18_)(>CPAF-C_2M_) **3**, (**f**) C_60_(>DPAF-C_18_)(>CPAF-C_2M_)_2_ **4**, in chloroform at a concentration of 1.0 × 10^−5^ M.

Optical absorption of **1** and **2** [Figure 6(d) and (c), respectively] was characterized by two distinguishable bands centered at 260 and 325–327 nm both arising from the C_60_> cage moiety that agrees with allowed ^1^T_1u_→^1^A_g_ transition bands of pristine C_60_ [29]. The third band with *λ*_max_ at either 411 or 501 nm for **1** or **2**, respectively, matches approximately with those of the corresponding precursor compound BrDPAF-C_18_ [Figure 6(a)] or BrCPAF-C_2M_ [Figure 6(b)]. These bands are in the characteristic photoresponsive wavelength range of DPAF-C_18_ or CPAF-C_2M_ antenna, respectively. When these two types of antenna were simultaneously attached to the same C_60_ in **3**, two absorption bands with *λ*_max_ (*ε*) at 413 (3.9 × 10^4^) and 494 nm (2.3 × 10^4^ L/mol-cm) were observed in the spectrum showing extinction coefficient *ε* values matching roughly with those of **1** and **2**. This clearly revealed a 1:1 ratio of DPAF-C_18_/CPAF-C_2M_ in **3** consistent with its composition. As the number of CPAF-C_2M_ antenna being increased to two in **4**, the corresponding two bands remained in the same range with *λ*_max_ (*ε*) at 417 (4.6 × 10^4^) and 500 nm (4.6 × 10^4^ L/mol-cm). The extinction coefficient *ε* value of the second band is nearly double to that of **3**. The structural modification resulted in approximately equal visible absorption in intensity over the full wavelength range of 400–550 nm. Accordingly, these bands can be utilized for the corresponding near-IR two-photon absorption excitation at 800–1,100 nm, giving broadband characteristics of the materials while exhibiting good linear transparency beyond 800 nm [Figure 6(e) and (f)]. In the long-wavelength absorption region beyond 650 nm, a very weak characteristic steady-state absorption band of methano[60]fullerene (C_60_>) moiety became noticeable at 695 nm only at an increased concentration of 4.5 × 10^−4^ M in CHCl_3_. 

**Figure 7:**
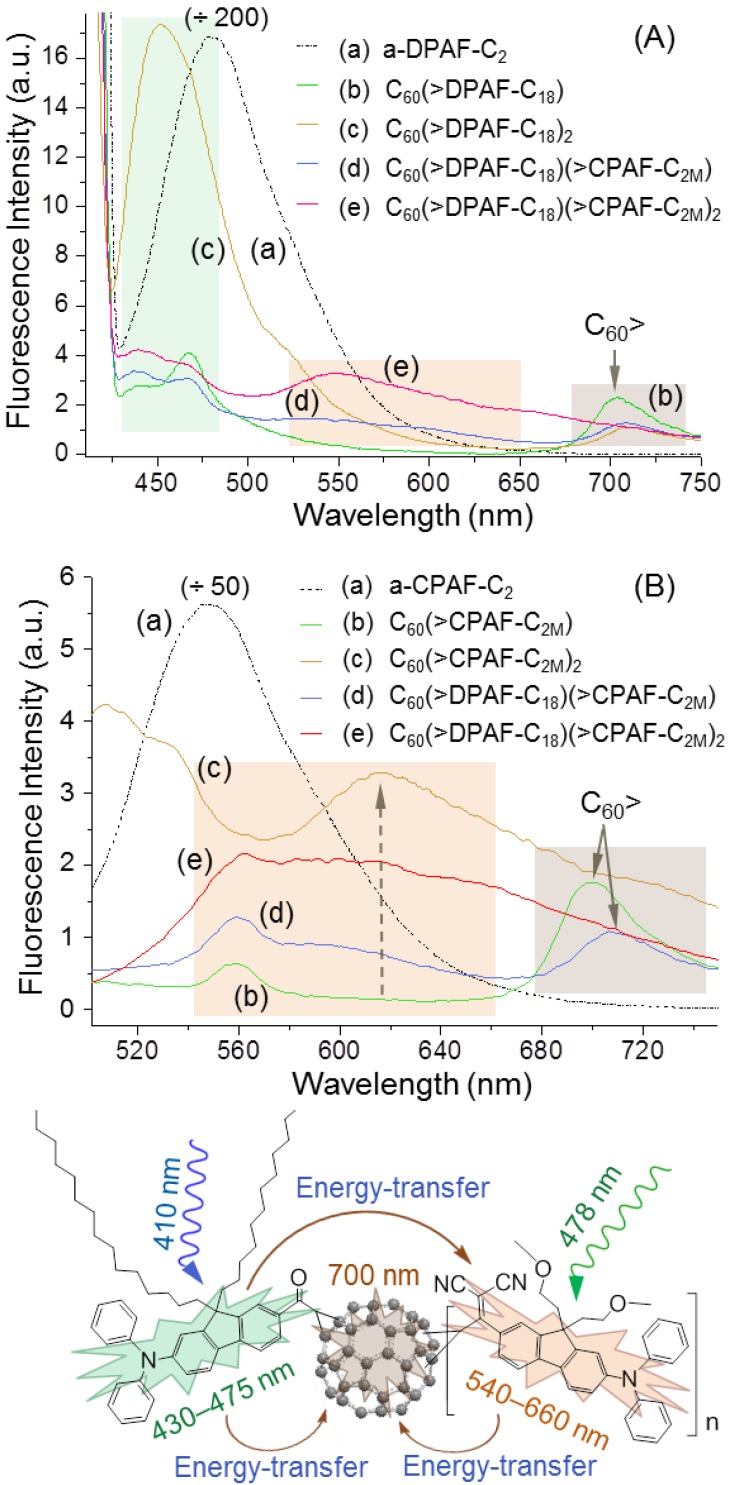
Steady-state fluorescence spectra of (**A**) (**a**) **10**, (**b**) **1** (including a Raman peak at 470 nm), (**c**) C_60_(>DPAF-C_18_)_2_, (**d**) **3**, and (**e**) **4** at the excitation wavelength of 410 nm and (**B**) (**a**) **11**, (**b**) **2** (including a Raman peak at 558 nm), (**c**) C_60_(>CPAF-C_2M_)_2_, (**d**) **3**, and (**e**) **4** at the excitation wavelength of 478 nm. The concentration of all samples was 1.0 × 10^−5^ M in toluene.

It is noteworthy that excited state intramolecular energy-transfer resonance phenomena between the DPAF-C_18_ and CPAF-C_2M_ antenna around the cage surface of **3** and **4** were observed. We first characterized the steady-state fluorescence (FL) emission of each antenna component using the model compound a-DPAF-C_2_ **10** and a-CPAF-C_2_ **11** (Scheme 1) in toluene as the spectroscopic reference. Upon photoexcitation of **10** at 410 nm to match with the optical absorption band of DPAF-C_18_, strong fluorescence emissions of ^1^(a-DPAF-C_2_)* centered at 481 nm (*λ*_max,em_) [Figure 7A(a)] were detected. Likewise, strong FL emissions of ^1^(a-CPAF-C_2_)* centered at 543 nm (*λ*_max,em_) [Figure 7B(a)] were observed when **11** was irradiated at 478 nm which matches with the optical absorption band of CPAF-C_2M_. As expected, highly efficient intramolecular fluorescence quenching of these two bands by C_60_ occurred when **1** and **2** were photoexcited at the same corresponding light wavelength, as shown in Figure 7A(b) and Figure 7B(b), respectively. This photophysical event led to the subsequent emission from the ^1^(C_60_>)* → ^1^(C_60_>)_o_ transition at 704 and 708 nm, respectively. The possible phosphorescence emission from ^3^(C_60_>)* → ^1^(C_60_>)_o_ transition expected at ~800–850 nm was too weak to be detected. In the case of the bisadduct C_60_(>DPAF-C_18_)_2_, two FL bands with *λ*_max_ at 451 and 525 (shoulder) nm [Figure 7A(c)] were shown, indicating incomplete quenching of C_60_[>^1^(DPAF)*-C_18_]_2_ by C_60_> when the number of antenna are more than one. Similarly, three fluorescence bands with *λ*_max_ at 506, 531, and 615 (broad) nm [Figure 7B(c)] were found for the bisadduct C_60_(>CPAF-C_2M_)_2_. Owing to high similarity on the structural moieties, these FL bands were used as the reference for the FL spectroscopic characterization of **3** and **4**. 

Interestingly, upon photoexcitation of the triad **3** specifically on the DPAF-C_18_ antenna moiety at 410 nm, the resulting FL spectrum [Figure 7A(d)] displayed a weak broad FL band at 448 [from ^1^(DPAF)*-C_18_] and broad bands at 525–650 nm along with the ^1^(C_60_>)* emission band centered at 708 nm. The latter broad bands fit in the similar range as those of C_60_[>^1^(CPAF)*-C_2M_]_2_. As the number of CPAF-C_2M_ antenna being increased by one to the structure of tetrad C_60_(>DPAF-C_18_)(>CPAF-C_2M_)_2_ **4**, the intensity of broad FL bands at 525–650 nm became more pronounced while retaining the same intensity of the ^1^(C_60_>)* emission band at 709 nm [Figure 7A(e)]. The data revealed intramolecular Förster energy-transfer resonance from the photoexcited C_60_[>^1^(DPAF)*-C_18_](>CPAF-C_2M_)_2_ state to both ^1^C_60_*(>DPAF-C_18_)(>CPAF-C_2M_)_2_ and C_60_(>DPAF-C_18_)[>^1^(CPAF)*-C_2M_]_2_ states. The latter energy-transfer is possible since: (i) the energy level of ^1^(CPAF)*-C_2M_ is lower than that of ^1^(DPAF)*-C_18_, (ii) the energy of this FL band at 430–475 nm is slightly higher than that of the CPAF-C_2M_ absorption *λ*_max_ at 500 nm, and (iii) there is a partial overlap of emission/absorption bands to enhance the energy-transfer efficiency. Conversely, photoexcitation of **3** specifically on the CPAF-C_2M_ antenna moiety at 478 nm, the resulting FL spectrum [Figure 7B(d)] showed only a weak broad FL band at 540–660 nm along with the ^1^(C_60_>)* emission band centered at 707 nm. Intensity of the former broad band was significantly increased using **4** [Figure 7B(e)] with photoexcitation on both two CPAF-C_2M_ antenna moieties. This confirmed the band was contributed from the C_60_(>DPAF-C_18_)[>^1^(CPAF)*-C_2M_]_2_ state, which was capable of inducing the ^1^C_60_*(>DPAF-C_18_)(>CPAF-C_2M_)_2_ state subsequently.

Data of femtosecond Z-scans and nonlinear light-intensity transmittance reduction measurements of C_60_(>CPAF-C_9_), **3**, and **4**, performed as a function of irradiance intensity using 125-fs laser pulses at either 780 nm (corresponding to the two-photon absorption of DPAF moieties) or 980 nm (corresponding to the two-photon absorption of CPAF moieties) at the concentration of 5 × 10^−3^ M in toluene, were provided in the supporting information. These data substantiated the nonlinear photonic characteristics of **3** and **4** showing dual NIR two-photon absorption capability that led to large nonlinear light-transmittance reduction in intensity in these two wavelength ranges up to the fs-laser fluence of 120 GW/cm^2^. Observed sufficiently large two-photon absorption cross-section values of **3** and **4** may allow their uses as the nanocarbon core of 2*γ*-PDT agents after the chemical modification with water-soluble side-chains and cationic targeting segments on the fluorene ring moiety.

## 3. Experimental

### 3.1. Materials

The reagents 1,8-diazabicyclo[5,4,0]-undec-7-ene (DBU), 1-bromooctadecane, 2-bromofluorene, sodium *t*-butoxide, potassium *t*-butoxide, aluminum chloride, titanium chloride, *rac*-BINAP, *tris*(dibenzylideneacetone)dipalladium(0), malononitrile, and 2-methoxy-ethanol, were purchased from Aldrich Chemicals (city, state abbrev, USA) and used without further purification. The chemical 1-bromooctadecane was purchased from Tokyo Chemical Industry Co., Ltd. (Waltham, MA, USA). A C_60_ sample with a purity of 99.0% was purchased from Term USA, Inc. (Fort Bragg, CA, USA). Both C_60_(>DPAF-C_18_)_2_ and C_60_(>CPAF-C_2M_)_2_ were synthesized by the similar methods described below.

### 3.2. Spectroscopic Measurements

Infrared spectra were recorded as KBr pellets on a Thermo Nicolet Avatar 370 FT-IR spectrometer. ^1^H-NMR and ^13^C-NMR spectra were recorded on a Bruker Avance Spectrospin-500 spectrometer. UV-vis spectra were recorded on a Perkin Elmer Lambda 750 UV-vis-NIR Spectrometer. Photoluminescence (PL) spectra were measured using PTI Fluorescence Master Systems connected with a photomultiplier (914 Photomultiplier Detection System) with Xenon short arc lamp as the excitation source. Mass spectroscopic measurements were performed by the use of positive ion matrix-assisted laser desorption ionization (MALDI–TOF) technique on a micromass M@LDI-LR mass spectrometer. The sample blended or dissolved in the matrix material was irradiated by nitrogen UV laser at 337 nm with 10 Hz pulses under high vacuum. Mass ion peaks were identified for the spectrum using the MassLynx v4.0 software. In a typical experiment, the samples of C_60_(>DPAF-C_18_), C_60_(>CPAF-C_2M_), C_60_(>DPAF-C_18_)(>CPAF-C_2M_), or C_60_(>DPAF-C_18_)(>CPAF-C_2M_)_2_ were dissolved in CHCl_3_ in a concentration of 1.0 mg/mL. The matrix of 3,5-dimethoxy-4-hydroxycinnamic acid (sinapic acid) was dissolved in THF in a concentration of 10 mg/mL. The solution of matrix (1.0 mL) was taken and mixed with the sample solution (0.1 mL) prior to the deposition on a stainless-steel MALDI target probe. It was subsequently dried at ambient temperature.

### 3.3. Synthetic Procedures

#### 3.3.1. Synthesis of 9,9-Di(2-methoxyethyl)-2-diphenylaminofluorene, DPAF-C_2M_ (**6**)

**Part A**: In a round-bottom flask containing a mixture of triethylamine (19.9 mL, 0.14 mol), 2-methoxyethanol (10.3 mL, 0.13 mol), and anhydrous dichloroethane (150 mL) at 0 °C was added methanesulfonyl chloride (11.1 mL, 0.14 mol) over a period of 20 min. The mixture was warmed to ambient temperature under a nitrogen atmospheric pressure and stirred for 12 h. It was quenched by the addition of water and washed with water (2 × 150 mL), dilute hydrochloric acid (1 × 100 mL), and saturated sodium bicarbonate (1 × 100 mL) in sequence. The organic layer was dried over sodium sulfate and concentrated *in vacuo*. The crude brownish liquid was vacuum distilled at 120−130 °C to afford 2-methoxyethylmethanesulfonate (17.9 g) in a nearly quantitative yield; ^1^H-NMR (CDCl_3_, ppm) *δ* 4.36 (t, *J* = 4.41 Hz, 2H), 3.66 (t, *J* = 4.41 Hz, 2H), 3.40 (s, 3H), and 3.05 (s, 3H).

**Part B**: In a round-bottom flask containing a mixture of 2-diphenylaminofluorene **5** (DPAF) (0.52 g, 1.56 mmol) and potassium *t*-butoxide (0.38 g, 3.39 mmol) in dry THF (30 mL) at 0 °C was added 2-methoxyethylmethanesulfonate (10.53 g, 3.4 mmol) over 10 min. The mixture was warmed to ambient temperature under a nitrogen atmosphere and stirred for 4.0 h. The reaction mixture was washed with brine (20 mL) and water (20 mL). Organic layer was dried over sodium sulfate and concentrated *in vacuo*. The crude product was then purified by column chromatography [silica gel, toluene−ethyl acetate (3:1) as the eluent] via a chromatographic fraction corresponding to *R*_f_ = 0.7 on TLC (SiO_2_) with the same eluent to afford DPAF-C_2M_ **6** as white solids in a yield of 94% (0.66 g). Spectroscopic data: MALDI-MS (TOF) *m/z* 449 calculated for ^12^C_31_^1^H_31_^14^N_1_^16^O_2_; found, *m/z* 450 (MH^+^); ^1^H-NMR (CDCl_3_, ppm) *δ* 7.61 (d, *J* = 7.60 Hz, 1H), 7.55 (d, *J* = 7.55 Hz, 1H), 7.38 (d, *J* = 7.37 Hz, 1H), 7.31 (t, *J* = 7.32 Hz, 1H), 7.28−7.24 (m, 5H), 7.18−7.10 (m, 6H), 7.13−7.00 (m, 2H), 3.04 (s, 6H), 2.79−2.71 (m, 4H), and 2.26−2.20 (m, 4H); ^13^C-NMR (CDCl_3_, ppm) *δ* 150.66, 149.32, 148.32, 147.95, 140.67, 135.72, 129.67, 127.79, 127.08, 124.43, 124.12, 123.18, 120.98, 119.69, 69.08, 58.77, 51.45, and 39.76.

#### 3.3.2. Synthesis of 7-*α*-Bromoacetyl-9,9-di(2-methoxyethyl)-2-diphenylaminofluorene, BrDPAF-C_2M_ (**7**)

To a suspension of aluminum chloride (4.8 g, 36 mmol) in 1,2-dichloroethane (200 mL) at 0 °C was added a solution of DPAF-C_2M_ **6** (5.44 g, 12.1 mmol) in 1,2-dichloroethane (50 mL). It was added *α*-bromoacetyl bromide (2.44 g, 12.1 mmol) over a period of 10 min. The mixture was stirred for 4.0 h at 0 °C. The solution was worked up by slow addition of dilute HCl (1.0 N) solution (50 mL) while maintaining the temperature at 0 °C. The resulting organic layer was washed subsequently with dilute brine (2 × 50 mL) and water (2 × 50 mL) at room temperature and dried over magnesium sulfate. It was followed by the solvent removal *in vacuo*. The crude products were purified by column chromatography [silica gel, hexane−ethyl acetate (4:1) as the eluent] at its chromatographic band corresponding to *R*_f_ = 0.2 on TLC (SiO_2_) with the same eluent to afford BrDPAF-C_2M_ **7** in 91% yield (6.3 g). Spectroscopic data: FT-IR (KBr) *ν*_max_ 3,054 (w), 3037 (w), 2,925 (m), 2,871 (m), 2,804 (w), 1,693(w), 1,673 (m), 1,593 (s), 1,490 (s), 1,467 (m), 1,430 (w), 1,388 (w), 1,320 (w), 1,279 (s), 1,194 (w), 1,113 (m), 1,026 (w), 820 (w), 754 (m), 697 (m), 669 (w), and 627 (w) cm^−1^; UV-vis (CHCl_3_) *λ*_max_ (*ε*) 299 (1.4 × 10^4^) and 407 (2.1 × 10^4^ L/mol-cm); ^1^H-NMR (CDCl_3_, ppm) *δ* 8.03−7.99 (m, 2H), 7.69 (d, *J* = 7.6 Hz, 1H), 7.63 (d, *J* = 7.5 Hz, 1H), 7.34−7.09 (m, 10H), 7.08−7.06 (m, 2H), 4.53 (s, 2H), 3.06 (s, 6H), 2.84−2.75 (m, 4H), and 2.33−2.21 (m, 4H); ^13^C-NMR (CDCl_3_) δ 190.99, 151.81, 149.32, 149.20, 147.42, 146.14, 132.83, 131.74, 129.38, 129.29, 124.66, 123.46, 122.80, 121.81, 119.06, 117.86, 68.52, 58.29, 51.30, 38.89, and 31.05.

#### 3.3.3. Synthesis of 7-*α*-Bromoacetyl-9,9-dioctadecyl-2-diphenylaminofluorene, BrDPAF-C_18_ (**8**)

**Part A**: In a round-bottom flask containing a mixture of 2-diphenylaminofluorene **5** (DPAF, 1.0 g, 3.0 mmol), potassium *t*-butoxide (1.0 g, 8.9 mmol) in dry THF (30 mL) at 0 °C was added 1-bromooctadecane (2.0 g, 6.0 mmol) over 10 min. The mixture was warmed to ambient temperature under a nitrogen atmosphere and stirred overnight. The reaction mixture was washed with brine (40 mL) and water (40 mL) in sequence. The organic layer was dried over sodium sulfate and concentrated *in vacuo*. The crude product was purified by column chromatography [silica gel, hexane−toluene (4:1) as the eluent] as a chromatographic fraction corresponding to *R*_f_ = 0.8 on TLC (SiO_2_) with the same eluent to give 9,9-dioctadecanyl-2-diphenylaminofluorene DPAF-C_18_ in 97% yield (2.44 g). Spectroscopic data: FT-IR (KBr) *ν*_max_ 3,067 (w), 3,036 (w), 2,924 (s), 2,853 (s), 1,599 (m), 1,494 (m), 1,451 (w), 1,331 (w), 1,277 (m), 1,154 (w), 1,075 (w), 1,029 (w), 824 (w), 751 (m), 737 (m), 696 (m), 623 (w), and 513 (w) cm^−1^; MALDI-MS (TOF) *m/*z 838 calculated for ^12^C_61_^1^H_91_^14^N_1_; found, *m/z* 839 (MH^+^); ^1^H-NMR (CDCl_3_, ppm) *δ* 7.63 (d, *J* = 6.9, 1H), 7.58 (d, *J* = 68.2, 1H), 7.33−7.25 (m, 7H), 7.15−7.13 (m, 5H), 7.05−7.01 (m, 3H), 1.93−1.81 (m, 4H), 1.33−1.07 (m, 60H), 0.91 (t, *J* = 6.94 Hz, 6H), and 0.73−0.62 (m, 4H); ^1^^3^C-NMR (CDCl_3_, ppm) *δ* 152.50, 148.45, 147.44, 141.29, 136.81, 129.52, 124.12, 123.98, 123.09, 122.78, 120.70, 119.93, and 119.49.

**Part B**: To a suspension of aluminum chloride (0.32 g, 2.4 mmol) in 1,2-dichloroethane (50 mL) at 0 °C was added a solution of DPAF-C_18_ (1.0 g, 1.2 mmol) in 1,2-dichloroethane (30 mL). It was then added by *α*-bromoacetyl bromide (0.30 g, 1.5 mmol) over 10 min. The mixture was stirred for 4.0 h at 0 °C. The solution was diluted by a slow addition of water (100 mL) while maintaining the reaction mixture temperature below 0 °C. The resulting organic layer was washed subsequently with dilute hydrochloric acid (1.0 N, 30 mL) and water (2 × 30 mL), and dried over magnesium sulfate followed by the solvent removal *in vacuo*. The crude yellow oil was purified by column chromatography [SiO_2_, hexane−EtOAc (19:1) as the eluent] to afford BrDPAF-C_18_ **8** in 96% yield (1.3 g). The product gave a chromatographic *R*_f_ at 0.5 on TLC (SiO_2_) with the same eluent. Spectroscopic data: FT-IR (KBr) *ν*_max_ 3,063 (w), 3,034 (w), 2,923 (s), 2,852 (s), 1,677 (m), 1,595 (m), 1,493 (m), 1,466 (w), 1,346 (w), 1,279 (m), 1,182 (w), 1,027 (w), 819 (w), 753 (w), 697 (m), 620 (w), and 508 (w) cm^−1^; UV-vis (CHCl_3_) *λ*_max_ (*ε*) 292 (1.9 × 10^4^) and 407 (2.5 × 10^4^ L/mol-cm); ^1^H-NMR (CDCl_3_, ppm) *δ* 7.95 (d,* J* = 8.18 Hz, 1H), 7.93 (s, 1H), 7.64 (d,* J* = 7.91 Hz, 1H), 7.59 (d,* J* = 8.23 Hz, 1H), 7.27−7.23 (m, 4H), 7.14−7.12 (m, 5H), 7.05−7.02 (m, 3H), 4.49 (s, 2H), 1.97−1.81 (m, 4H), 1.25−1.04 (m, 66H), 0.87 (t, *J* = 6.78 Hz, 6H), and 0.72−0.55 (br, 4H); ^13^C-NMR (CDCl_3_) *δ* 190.99 (**-C**=O), 153.63 (aminoaryl carbon), 151.06 (aminoaryl carbon), 148.81 (aminoaryl carbon), 147.61, 146.89, 133.96, 131.55, 129.25, 128.80, 124.36, 123.09, 122.78, 121.61, 118.82, 118.20, 55.23, 39.96, 31.90, 31.15, 29.90, 29.67, 29.64, 29.62, 29.57, 29.55, 29.34, 29.29, 23.83, 22.67, and 14.10.

#### 3.3.4. Synthesis of 7-(1,2-Dihydro-1,2-methano[60]fullerene-61-carbonyl)-9,9-dioctadecyl-2-diphenylaminofluorene, C_60_(>DPAF-C_18_) (**1**)

To a mixture of C_60_ (0.75 g, 1.1 mmol) and 7-*α*-bromoacetyl-9,9-dioctadecanyl-2-diphenylaminofluorene (BrDPAF-C_18_, **8**, 0.85 g, 1.1 mmol) in dry toluene (500 mL) was added DBU (0.18 ml, 1.2 mmol) under a nitrogen atmosphere. After stirring at room temperature for 5.0 h, suspended solids of the reaction mixture were filtered off and the filtrate was concentrated to a 10% volume. Crude product was precipitated by the addition of methanol and isolated by centrifugation (8000 rpm, 20 min). The isolated solid was found to be a mixture of the monoadduct and its bisadducts. Separation of these two product fractions were made by column chromatography (silica gel) using a solvent mixture of hexane−toluene (3:2) as the eluent. The first chromatographic band corresponding to *R*_f_ = 0.7 on TLC (SiO_2_, hexane-toluene, 3:1) afforded C_60_(>DPAF-C_18_) **1** as brown solids (1.12 g, 65% yield based on recovered C_60_). Spectroscopic data: FT-IR (KBr) *ν*_max_ 3,440 (m), 2,920 (s), 2,849 (s), 1,674 (-C=O, m), 1,632 (m), 1,593 (s), 1,491 (m), 1,463 (m), 1,427 (m), 1,346 (w), 1,331 (w), 1,316 (w), 1,273 (m), 1,239 (w), 1,200 (m), 1,186 (w), 1157 (w), 1028 (w), 817 (w), 752 (m), 738 (w), 696 (m), 575 (w), 547 (w), 526 (m), and 490 (m) cm^−1^; MALDI-MS (TOF) *m/z* 1598 calculated for ^12^C_123_^1^H_91_^14^N_1_^16^O_1_; found, *m/z* 1,601, 1,600 (MH^+^), 1,599, 866, 839, 762, 734, and 720; UV-vis (CHCl_3_) *λ*_max_ (*ε*) 260 (1.3 × 10^5^), 325 (4.7 × 10^4^), and 411 (3.6 × 10^4^ L/mol-cm) ^1^H-NMR (CDCl_3_, ppm) *δ* 8.43 (d, *J* = 6.9 Hz, 1H), 8.32 (s, 1H), 7.78 (d, *J* = 8.0 Hz, 1H), 7.61 (d, *J* = 8.0 Hz, 1H), 7.25−7.22 (m, 4H), 7.11−7.09 (m, 5H), 7.03−7.00 (m, 3H), 5.66 (s, 1H), 2.03−1.84 (m, 4H), 1.29−1.04 (m, 58H), 0.87 (t, *J* = 6.88 Hz , 6H), and 0.69 (br, 4H). ^13^C-NMR (CDCl_3_) *δ* 188.33 (**-C**=O), 153.55 (aminoaryl carbon), 151.20 (aminoaryl carbon), 148.77 (aminoaryl carbon), 147.96 (2C), 147.30 (2C), 147.20 (C), 146.73 (2C), 145.35 (2C), 145.24 (2C), 145.06 (2C), 144.96 (4C), 144.85 (2C), 144.70 (C), 144.52 (2C), 144.43 (4C), 144.13 (2C), 143.74 (2C), 143.49 (2C), 143.14 (2C), 142.96 (C), 142.91 (C), 142.83 (2C), 142.76 (2C), 142.57 (2C), 142.32 (2C), 142.07 (2C), 142.00 (2C), 141.90 (2C), 141.06 (2C), 140.76 (2C), 139.36 (2C), 136.46 (2C), 133.57, 133.22, 129.22, 128.62, 124.40, 123.15, 122.83, 122.42, 121.71, 119.14, 117.78, 72.48 (fullerenyl sp^3^ carbons), 55.09, 44.58, 40.14 (cyclopropanyl C_60_> carbon), 32.00, 30.16, 29.81, 29.56, 29.47, 24.11, 22.87, and 14.22. A total of 30 carbon peaks were accounted for 58 fullerenyl sp^2^ carbons at *δ* 136–148 indicated a *C*_2_-symmetry of the fullerene cage.

#### 3.3.5. Synthesis of 7-[2-Bromo-1-(1,1-dicyanoethylenyl)-1-methyl]-9,9-di(2-dimethoxyethyl)-2-diphenylaminofluorene, BrCPAF-C_2M_ (**9**)

To a mixture of 7-*α*-bromoacetyl-9,9-di(2-methoxyethyl)-2-diphenylaminofluorene (BrDPAF-C_2M_, 7, 2.17 g, 3.8 mmol) and malononitrile (0.29 g, 4.4 mmol) in dry chloroform (100 mL) was added pyridine (3.0 mL) while stirring under a nitrogen atmosphere. To this solution, titanium tetrachloride (1.0 mL, excess) was added in. After stirring at room temperature for 5.0 min, the reaction mixture was quenched with water (90 mL). The resulting organic layer was washed several times with water (100 mL each), dried over magnesium sulfate, and concentrated *in vacuo* to afford the crude orange-red oil. It was purified on a preparative chromatographic plate (PTLC, SiO_2_, CHCl_3_ as the eluent). A product fraction collected at *R*_f_ = 0.6 [toluene−ethyl acetate (4:1) as the eluent] gave BrCPAF-C_2M_**9** in 89% yield (2.1 g). Spectroscopic data: FT-IR (KBr) *ν*_max_ 3,058 (w), 3,035 (w), 2,924 (m), 2,870 (m), 2,855 (m), 2,809 (w), 2,226 (m), 1,593 (s), 1,547 (m), 1,490 (s), 1,468 (m), 1,451 (w), 1,384 (w), 1,346 (m), 1,318 (m), 1,279 (s), 1,191 (w), 1,113 (m), 1,028 (w), 957 (w), 821 (w), 755 (m), 698 (m), and 511 (w) cm^−1^; UV-vis (CHCl_3_) *λ*_max_ (*ε*) 316 (2.1 × 10^4^) and 489 (1.7 × 10^4^ L/mol-cm); ^1^H-NMR (CDCl_3_, ppm) *δ* 7.70−7.66 (m, 3H), 7.59 (d, *J* = 8.3 Hz, 1H), 7.30−7.27 (m, 4H), 7.15−7.13 (m, 5H), 7.09−7.06 (m, 3H), 4.61 (s, 2H), 3.03 (s, 6H), 2.87−2.77 (m, 4H), and 2.32−2.18 (m, 4H); ^13^C-NMR (CDCl_3_) *δ* 170.85 [–**C**=C(CN)_2_], 151.77 (aminoaryl carbon), 149.91 (aminoaryl carbon), 149.38 (aminoaryl carbon), 147.28, 145.72, 132.39, 130.13, 129.36, 127.92, 124.74, 123.54, 122.91, 122.55, 121.72, 119.60, 117.55, 112.98 (–**C**≡N), 112.11 (–**C**≡N), 84.48 [=**C**(CN)_2_], 68.42, 58.31, 51.64, 38.93, and 28.68.

#### 3.3.6. Synthesis of 7-(1,2-Dihydro-1,2-methano[60]fullerene-61-{1,1-dicyanoethylenyl})-9,9-di(2-methoxyethyl)-2-diphenylaminofluorene, C_6_0(>CPAF-C_2M_) (**2**)

To a mixture of C_60_ (0.18 g, 0.25 mmol) and 7-[2-bromo-1-(1,1-dicyanoethylenyl)-1-methyl]-9,9-di(2-methoxyethyl)-2-diphenylaminofluorene (BrCPAF-C_2M_, **9**, 0.15 g, 0.24 mmol) in dry toluene (150 mL) was added 1,8-diazabicyclo[5,4,0]-undec-7-ene (DBU, 0.1 M, 2.6 mL) under a nitrogen atmosphere. After stirring at room temperature for a period of 5.0 h, the reaction mixture was concentrated to a volume of approximately 10 mL. Crude product was precipitated by the addition of methanol and isolated by centrifugation (8,000 rpm, 20 min). The precipitate was further purified by column chromatography [silica gel, toluene−ethyl acetate (4:1) as the eluent] at the corresponding chromatographic *R*_f_ = 0.8 on TLC (SiO_2_) with the same eluent to afford C_60_(>CPAF-C_2M_) **2** in 53% yield (0.16 g). Spectroscopic data: FT-IR (KBr) *ν*_max_ 3,439 (s), 2,980 (w), 2,920 (m), 2,868 (m), 2,824 (w), 2798 (w), 2,224 (–C≡N, m), 1,629 (m), 1,594 (vs), 1,538 (m), 1,491 (m), 1,466 (m), 1,428 (m), 1,347 (m), 1,319 (m), 1,279 (s), 1,186 (m), 1,115 (m), 1,028 (w), 958 (w), 888 (w), 820 (m), 805 (w), 754 (m), 697 (m), 668 (w), 577 (w), and 527 (m) cm^−1^; MALDI-MS (TOF) *m/z* 1,257 calculated for ^12^C_96_^1^H_31_^14^N_3_^16^O_2_; found, *m/z* 1,260, 1,259, 1,258 (MH^+^), 1,155, 987, 965, 919, 735, and 720; UV-vis (CHCl_3_) *λ*_max_ (*ε*) 260 (1.2 × 10^5^), 327 (5.1 × 10^4^), and 501 nm (1.7 × 10^4^ L/mol-cm); ^1^H-NMR (CDCl_3_, ppm) *δ* 8.14 (d, *J* = 8.2 Hz, 1H), 8.11 (s, 1H), 7.76 (d, *J* = 7.97 Hz, 1H), 7.56 (d, *J* = 7.97 Hz, 1H), 7.26−7.23 (m, 4H), 7.09−7.08 (m, 5H), 7.06−7.03 (m, 3H), 5.51 (s, 1H), 2.95 (s, 6H), 2.73 (t, *J* = 7.97 Hz, 4H), and 2.32−2.18 (m, 4H); ^13^C-NMR (CDCl_3_) *δ* 167.64 [–**C**=C(CN)_2_], 151.83 (aminoaryl carbon), 150.31 (aminoaryl carbon), 149.45 (aminoaryl carbon), 147.23 (2C), 147.06 (2C), 146.07 (C), 145.69 (2C), 145.25 (4C), 145.21 (2C), 145.18 (2C), 145.15 (2C), 145.03 (2C), 144.72 (4C), 144.68 (4C), 144.53 (2C), 144.40 (2C), 144.15 (C), 143.69 (2C), 143.62 (2C), 142.94 (2C), 142.89 (4C), 142.36 (2C), 141.96 (2C), 141.94 (2C), 141.83 (2C), 141.36 (2C), 140.97 (2C), 137.31 (2C), 136.95 (2C), 132.12, 129.36, 128.46, 124.76, 123.63, 122.89, 122.39, 121.89, 119.67, 117.47, 112.99 (–**C**≡N), 112.92, 88.07 [=**C**(CN)_2_], 72.30 (fullerenyl sp^3^ carbons), 68.33, 58.26, 51.56, 41.22 (cyclopropanyl C_60_> carbon), and 39.26. A total of 30 carbon peaks representing 58 fullerenyl sp^2^ carbons at *δ* 136–148 indicated a *C*_2_-symmetry of the fullerene cage. 

#### 3.3.7. Synthesis of Hybrid [(9,9-Dioctadecyl-2-diphenylaminofluorenyl)-7-carbonyl]-{[9,9-(2-dimethoxyethyl)-2-diphenylaminofluorenyl]-7-(1,1-dicyanoethylenyl)}-bis(1,2-dihydro-1,2-methano)-[60]fullerenyl Triad C_60_(>DPAF-C_18_)(>CPAF-C_2M_) (**3**) and its Tetrad Analogous C_60_(>DPAF-C_18_) (>CPAF-C_2M_)_2_ (**4**)

To the mixture of 7-(1,2-dihydro-1,2-methano[60]fullerene-61-carbonyl)-9,9-di(octadecyl)-2-diphenylaminofluorene C_60_(>DPAF-C_18_) **1** (0.48 g, 0.3 mmol) and 7-[2-bromo-1-(1,1-dicyanoethylenyl)-1-methyl]-9,9-di(2-methoxyethyl)-2-diphenylaminofluorene (BrCPAF-C_2M_, **9**, 0.37 g, 0.6 mmol) in dry toluene (100 mL) was added 1,8-diazabicyclo[5,4,0]-undec-7-ene (DBU, 0.1 M, 6.0 mL) slowly under a nitrogen atmosphere. After stirring at room temperature for a period of 5.0 h, the reaction mixture was concentrated to a volume of approximately 10 mL. Crude product was precipitated by the addition of methanol and isolated by centrifugation (8000 rpm, 20 min). The isolated solid was found to be a mixture of the fullerene multiadducts. Separation of these mixture was made by column chromatography (silica gel) using a solvent mixture of toluene–ethyl acetate (9:1) as the eluent. The first chromatographic band gave the unreacted starting compound **1** (0.08 g, 0.05 mmol). The second chromatographic band corresponding to *R*_f_ = 0.5 on the thin-layer chromatographic plate [TLC, SiO_2_, toluene–ethyl acetate (9:1) as the eluent] afforded the bisadduct product C_60_(>DPAF-C_18_)(>CPAF-C_2M_) **3** as orange-brown solids (0.15 g, 0.07 mmol) in a 28% yield [based on the recovered C_60_(>DPAF-C_18_) amount]. The third chromatographic band corresponding to *R*_f_ = 0.4 on the thin-layer chromatographic plate [TLC, SiO_2_, toluene-ethyl acetate (4:1) as the eluent] afforded the trisadduct product C_60_(>DPAF-C_18_)(>CPAF-C_2M_)_2_ **4** as red-brown solids (0.28 g, 0.10 mmol) in a yield of 40% [based on the recovered C_60_(>DPAF-C_18_) amount]. Spectroscopic data of C_60_(>DPAF-C_18_)(>CPAF-C_2M_) **3**: FT-IR (KBr) *ν*_max_ 3,424 (w), 3,063 (w), 3,030 (w), 2,921 (m), 2,850 (m), 2,223 (m), 1,678 (m), 1,593 (s), 1,568 (m), 1,537 (w), 1,492 (m), 1,465 (m), 1,426 (m), 1,346 (m), 1,277 (s), 1,202 (m), 1,115 (m), 1,074 (w), 962 (w), 895 (w), 819 (m), 753 (s), 696 (s), 578 (w), 526 (m), and 491 (w) cm^−1^; MALDI-MS (TOF) *m/z* 2,135 calculated for ^12^C_159_^1^H_122_^14^N_4_^16^O_3_; found, *m/z* 2,136 (MH^+^), 2,135 (M^+^), 965, 920, 866, 763 {C_60_[>(C=O)-H]H^+^}, 735 (C_60_>H^+^), and 720 (C_60_^+^); UV-vis (CHCl_3_) *λ*_max_ (*ε*) 255 (1.1 × 10^5^), 304 (7.5 × 10^4^), 413 (3.9 × 10^4^), and 494 nm (2.3 × 10^4^ L/mol-cm); ^1^H-NMR (CDCl_3_, ppm) *δ* 8.51−7.52 (m, 8H), 7.28−7.22 (m, 8H), 7.14−7.00 (m, 16H), 5.76−5.18 (m, 2H), 3.01−2.90 (br, 6H), 2.82−2.67 (br, 4H), 2.40−2.14 (br, 4H), 2.06−1.80 (br, 4H), 1.31−1.04 (m, 58H), 0.87 (t, *J* = 6.72 Hz , 6H), and 0.67 (br, 4H). Spectroscopic data of C_60_(>DPAF-C_18_)(>CPAF-C_2M_)_2_ **4**: FT-IR (KBr) *ν*_max_ 3,433 (m), 3,060 (w), 3,027 (w), 2,922 (m), 2,851 (m), 2,224 (m), 1,679 (w), 1,594 (s), 1,539 (w), 1,492 (s), 1,466 (m), 1,426 (m), 1,346 (m), 1,331 (w), 1,279 (s), 1,207 (w), 1,115 (m), 1028 (w), 958 (w), 892 (w), 818 (m), 753 (m), 697 (m), 616 (w), and 525 (w) cm^−1^; MALDI-MS (TOF) *m/z* 2,672 calculated for ^12^C_195_^1^H_153_^14^N_7_^16^O_5_; found, *m/z* 2,673 (MH^+^), 2,672 (M^+^), 965, 920, 866, 763 {C_60_[>(C=O)-H]H^+^}, 735 (C_60_>H^+^), and 720 (C_60_^+^); UV-vis (CHCl_3_) *λ*_max_ (*ε*) 304 (1.1 × 10^5^), 417 (4.6 × 10^4^), and 500 nm (4.6 × 10^4^ L/mol-cm); ^1^H-NMR (CDCl_3_, ppm) *δ* 8.44−7.46 (m, 16H), 7.09−7.03 (br, 32H), 5.57−4.72 (m, 3H), 3.08−2.86 (br, 12H), 2.73 (br, 8H), 2.21 (br, 8H), 1.91 (br, 4H), 1.3−0.95 (m, 58H), 0.86 (t, *J* = 6.55 Hz , 6H), and 0.64 (br, 4H).

## 4. Conclusions

Two new C_60_-(antenna)_x_ analogous compounds as branched triad C_60_(>DPAF-C_18_)(>CPAF-C_2M_) and tetrad C_60_(>DPAF-C_18_)(>CPAF-C_2M_)_2_ nanostructures were synthesized and characterized by various spectroscopic methods. The design of 2PA-responsive chromophores was made by covalently attaching multiple light-harvesting donor antenna units on a C_60_ (acceptor) via a periconjugation linkage within a separation distance of 2.6–3.5 Ǻ. This structural design was intended to facilitate the ultrafast femtosecond intramolecular energy-transfer process from the photoexcited C_60_[>^1^(DPAF)*-C_18_](>CPAF-C_2M_)_1or2_ or C_60_(>DPAF-C_18_)[>^1^(CPAF)*-C_2M_]_1or2_ to the C_60_ cage moiety upon two-photon pumping at either 780 or 980 nm, respectively. Interestingly, by adjustment of a higher number of CPAF-C_2M_ antenna, the resulting tetrads showed nearly equal absorption in extinction coefficients over the wavelength range of 400–550 nm that corresponds to near-IR two-photon based excitation wavelengths at 780–1,100 nm for broadband 2*γ*-PDT applications. We also found that the unique feature of intramolecular Förster energy-transfer phenomena from the photoexcited high-energy DPAF-C_18_ antenna unit to the low-energy CPAF-C_2M_ moiety at the fullerene cage surface gave the fluorescence emission at slightly longer wavelengths than 600 nm in a cascade fashion. It may be correlated to and provide an interesting mechanism for the enhancement of 2PA cross-section values of these hybrid C_60_-(antenna)_x_ nanostructures.

## Supplementary Materials

Supplementary materials can be accessed at: http://www.mdpi.com/1420-3049/18/8/9603/s1.

## References

[1] Guldi D.M., Prato M. (2000). Excited-state properties of C_60_ fullerene derivatives. Acc. Chem. Res..

[2] Fujitsuka M., Ito O., Nalwa H.S. (2004). Encyclopedia of Nanoscience and Nanotechnology.

[3] Bhawalkar J.D., Kumar N.D., Zhao C.-F., Prasad P.N. (1997). Two-photon photodynamic therapy. J. Clin. Laser Med. Surg..

[4] Brown S. (2008). Photodynamic Therapy: Two photons are better than one. Nat. Photonics.

[5] Spangler C.W., Starkey J.R., Dubinina G., Fahlstromb C., Shepard J. (2012). Optimization of targeted two-photon PDT triads for the treatment of head and neck cancers. Proc. SPIE.

[6] Spangler C.W., Starkey J., Rebane A., Drobizhev M., Meng F., Gong A. (2008). Synthesis, characterization and two-photon PDT efficacy studies of triads incorporating tumor targeting and imaging components. Proc. SPIE.

[7] Dahlstedt Z.E., Collins H.A., Balaz M., Kuimova M.K., Khurana M., Wilson B.C., Phillips D., Anderson H.L. (2009). One- and two-photon activated phototoxicity of conjugated porphyrin dimers with high two-photon absorption cross sections. Org. Biomol. Chem..

[8] Riggs J.E., Sun Y.-P. (1999). Optical limiting properties of [60]fullerene and methano[60]fullerene in solution *versus* in polymer matrix: the role of bimolecular processes and a consistent nonlinear absorption mechanism. J. Phys. Chem. A.

[9] Maggini M., Faveri C.D., Scorrano G., Prato M., Brusatin G., Guglielmi M., Meneghetti M., Signorini R., Bozio R. (1999). Synthesis and optical-limiting behavior of hybrid inorganic–organic materials from the sol–gel processing of organofullerenes. Chem. Eur. J..

[10] Chiang L.Y., Padmawar P.A., Canteewala T., Tan L.-S., He G.S., Kanna R., Vaia R., Lin T.-C., Zheng Q., Prasad P.N. (2002). Synthesis of C_60_-diphenylaminofluorene dyad with large 2PA cross-sections and efficient intramolecular two-photon energy transfer. Chem. Commun..

[11] Koudoumas E., Konstantaki M., Mavromanolakis A., Couris S., Fanti M., Zerbetto F., Kordatos K., Prato M. (2003). Large enhancement of the nonlinear optical response of reduced fullerene derivatives. Chem. Eur. J..

[12] Padmawar P.A., Canteenwala T., Verma S., Tan L.-S., Chiang L.Y. (2004). Synthesis and photophysical properties of C_60_-diphenylaminofluorene dyad and multiads. J. Macromol. Sci. A Pure Appl. Chem..

[13] Padmawar P.A., Canteenwala T., Tan L.-S., Chiang L.Y. (2006). Synthesis and characaterization of two-photon absorbing diphenylaminofluorenocarbonyl-methano[60]fullerenes. J. Mater. Chem..

[14] Padmawar P.A., Rogers J.O., He G.S., Chiang L.Y., Canteenwala T., Tan L.-S., Zheng Q., Lu C., Slagle J.E., Danilov E. (2006). Large cross-section enhancement and intramolecular energy transfer upon multiphoton absorption of hindered diphenylaminofluorene-C_60_ dyads and triads. Chem. Mater..

[15] Kopitkovas G., Chugreev A., Nierengarten J.F., Rio Y., Rehspringer J.L., Honerlage B. (2004). Reverse saturable absorption of fullerodendrimers in porous SiO_2_ sol-gel matrices. Opt. Mater..

[16] He G.S., Tan L.-S., Zheng Q., Prasad P.N. (2008). Multiphoton absorbing materials: molecular designs, characterizations, and applications. Chem. Rev..

[17] Spangler C.W. (1999). Recent development in the design of organic materials for optical power limiting. J. Mater. Chem..

[18] Mckay T.J., Staromlynska J., Wilson P., Davy J. (1999). Nonlinear luminescence in a Pt: ethynyl compound. J. Appl. Phys..

[19] Perry J.W., Nalwa H.S., Miyata S. (1997). Nonlinear Optics of Organic Molecules and Polymers.

[20] MacMahon S., Fong II R., Baran P.S., Safonov I., Wilson S.R., Schuster D.I. (2001). Synthetic approaches to a variety of covalently linked porphyrin-fullerene hybrids. J. Org. Chem..

[21] Li K., Schuster D.I., Guldi D.M., Herranz M.A., Echegoyen L. (2004). Convergent synthesis and photophysics of [60]fullerene/porphyrin-based rotaxanes. J. Am. Chem. Soc..

[22] Huang Y.Y., Sharma S.K., Dai T., Chung H., Yaroslavsky A., Garcia-Diaz M., Chang J., Chiang L.Y., Hamblin M.R. (2012). Can nanotechnology potentiate photodynamic therapy?. Nanotechnol. Rev..

[23] Sperandio F.F., Gupta A., Wang M., Chandran R., Sadasivam M., Huang Y.-Y., Chiang L.Y., Hamblin M.R. (2013). Photodynamic Therapy Mediated by Fullerenes and Their Derivatives.

[24] Elim H.I., Jeon S.-H., Verma S., Ji W., Tan L.-S., Urbas A., Chiang L.Y. (2008). Nonlinear optical transmission properties of C_60_ dyads consisting of a light-harvesting diphenylaminofluorene antenna. J. Phys. Chem. B.

[25] Chiang L.Y., Padmawar P.A., Rogers–Haley J.E., So G., Canteenwala T., Thota S., Tan L.-S., Pritzker K., Huang Y.-Y., Sharma S.K. (2010). Synthesis and characterization of highly photoresponsive fullerenyl dyads with a close chromophore antenna–C_60_ contact and effective photodynamic potential. J. Mater. Chem..

[26] Elim H.I., Anandakathir R., Jakubiak R., Chiang L.Y., Ji W., Tan L.S. (2007). Large concentration-dependent nonlinear optical responses of starburst diphenylaminofluorenocarbonyl methano[60]fullerene pentaads. J. Mater. Chem..

[27] El-Khouly M.E., Padmawar P., Araki Y., Verma S., Chiang L.Y., Ito O. (2006). Photoinduced processes in a tricomponent molecule consisting of diphenylaminofluorene-dicyanoethylene-methano[60]fullerene. J. Phys. Chem. A.

[28] Luo H., Fujitsuka M., Araki Y., Ito O., Padmawar P., Chiang L.Y. (2003). Inter- and intramolecular photoinduced electron-transfer processes between C_60_ and diphenylaminofluorene in solutions. J. Phys. Chem. B.

[29] Saito S., Oshiyama A. (1991). Cohesive mechanism and energy bands of solid C_60_. Phys. Rev. Lett..

