# Supplementary material for: Synthesis of Photoresponsive Dual NIR Two-Photon Absorptive [60]Fullerene Triads and Tetrads
Source: Molecules. 2013 Aug 12;18(8):9603–22. doi: 10.3390/molecules18089603 (PMC3830959; doi:10.3390/molecules18089603)
Supplement: Supplementary file 1 [file molecules-18-09603-s001.pdf]

Supplementary Information

S1. Spectroscopic Data

Spectroscopic characterization of **1** and **2** was performed mainly by (i) the clear detection of a group of molecular mass ion peaks with the maximum peak intensity centered at *m/z* 1600 (MH^+ of **1**) and 1258 (MH^+ of **2**) (Figure S1) using positive ion matrix-assisted laser desorption ionization (MALDI–TOF) mass spectroscopy and (ii) analyses of ^{13}C NMR spectra. The former spectra were also accompanied with two groups of fragmented mass ion peaks at *m/z* 720 and 734/735 corresponding to the mass units of C_{60} and C_{60}>, respectively, indicating high stability of the fullerene cage under MALDI-MS conditions.

Figure S1. MALDI-TOF mass spectra of (**a**) C_{60}(>CPAF-C_{2M}) and (**b**) C_{60}(>DPAF-C_{18}).

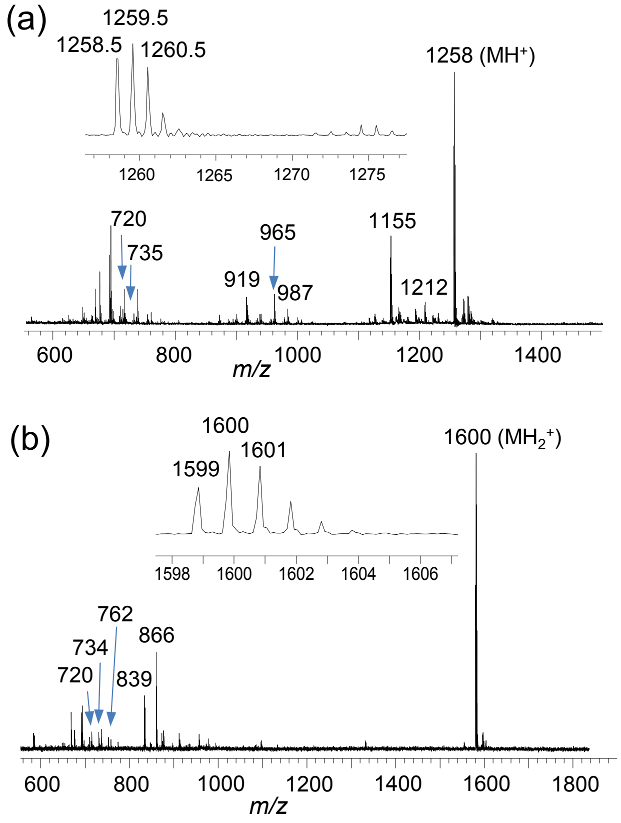

S2. Nonlinear Optical Data

S2.1. Z-Scan and Light-Intensity-Dependent Transmittance Measurements

Z-scan measurements and irradiance-dependent transmission measurements were carried out at the wavelength of either 780 or 980 nm using 125-fs laser pulses with the repetition rate of 1.0 kHz. Laser pulses were generated by an optical parametric amplifier system (TOPAS) pumped by a Ti-Sapphire regenerative amplifier (Spitfire Pro, Spectra Physics/Newport) and focused onto a 1.0-mm thick quartz cuvette containing a solution of methano[60]fullerene derivatives. Incident and transmitted laser intensities were monitored as the cuvette was moved (or *Z*-scanned) along the propagation direction of the laser pulses. The data sets were normalized to the linear transmittance and sample inhomogeneities

for all Z-scans by the correction of the background transmittance, $T(|Z| - Z_o)$. Total absorption was described by the change in the absorption coefficient $\Delta\alpha = \beta I$, where β and I are the 2PA coefficient and the light intensity, respectively. The absorption coefficient could be extracted from the line fitting between the Z-scan theory[1] and the data. The 2PA cross-section value was then calculated from the coefficient by the formula $\sigma_2 = \beta\hbar\omega/N$, where $\hbar\omega$ is the photon energy and N is the number of the molecules.

Scheme S1. The structure of C_{60}-(antenna)$_x$ (x = 1 or 2) monoadducts, hybrid bisadducts, and hybrid trisadducts.

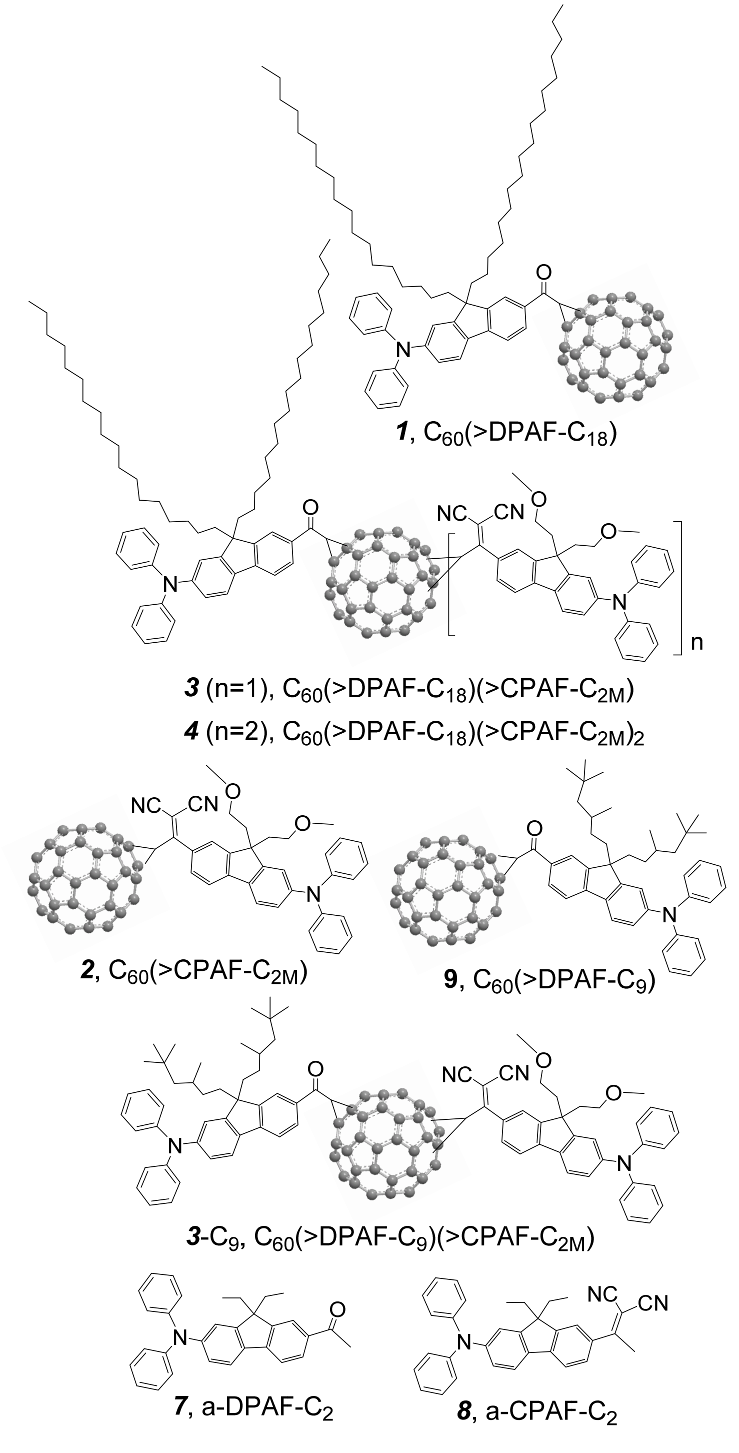

SB.2. Two-Photon Absorption (2PA) by Both Z-Scans and Light-Intensity-Dependent Transmittance Measurements

Simultaneous 2PA cross-sections (σ_2) and nonlinear light-transmittance measurements of **3** and **4** were carried out in toluene at wavelengths of either 780 or 980–1000 nm with the collection of femtosecond *Z*-scan data to demonstrate the nonlinear photoresponse ability of hybrid C_{60}-(antenna)$_{2–3}$ analogous triads and tetrads at different near-IR wavelength ranges. A C_{60}-(antenna)$_x$ nanostructure showing large 2PA-active NLO properties at many wavelengths or a continuous wavelength spectrum will facilitate their uses as broadband NLO materials. In the molecular structure of **3** and **4**, all chromophore components are 2PA-responsive that allows the corresponding branched C_{60}-(antenna)$_x$ (x = 2–3) nanomaterials to exhibit appreciable 2PA cross-sections at three adjacent spectral ranges of 600–750 (based on C_{60}> moiety), 740–900 (based on DPAF-C_{18} moiety), and 860–1100 (based on CPAF-C_{2M} moiety) nm. These three ranges correspond to the strong linear optical absorption band of the C_{60}> cage at 280–375 nm, the DPAF-C_{18} antenna at 370–450 nm, and the CPAF-C_{2M} antenna at 430–550 nm. By tuning the ratio of CPAF-C_{2M}/DPAF-C_{18} to two, a nearly even linear absorption extinction coefficient over 400–520 nm was achieved for creating the corresponding broadband 2PA features over 800–1050 nm with no apparent absorption minimum.

The use of fs pulses eliminates potential accumulative thermal scattering effects in the material environment. A pulse duration width of 125 fs was used to focus the study primarily on the 2PA process of each antenna. This pulse width matches with the value of 130 fs required for the effective intramolecular energy-transfer from the excited antenna to the C_{60}> moiety, as discussed above. Therefore, the measured σ_2 values at 125 fs should not cover the excited singlet state absorption (S_1–S_n) of $^1C_{60}$*(>DPAF-C_{18})(>CPAF-C_{2M})$_{1or2}$ cage moiety. As a result, open-aperture *Z*-scan data of two hybrid triads C_{60}(>DPAF-C_9)(>CPAF-C_{2M}) and C_{60}(>DPAF-C_{18})(>CPAF-C_{2M}) **3** and one hybrid tetrad C_{60}(>DPAF-C_{18})(>CPAF-C_{2M})$_2$ **4** at 780–800 and 980 nm in toluene under different conditions were shown in Figure S2 with all parameters summarized in Table S1. For the comparison and discussion purpose, the σ_2 values of C_{60}(>CPAF-C_9) and C_{60}(>DPAF-C_9) were also included. The method for data reduction, line fitting, and 2PA cross-section calculation was described in the experimental section.

We used the sample of C_{60}(>CPAF-C_9) to demonstrate the strong concentration-dependent σ_2 values through solution concentration between 10^{-3}–10^{-2} M (Table S1) owing to the aggregation effect. Only a moderate σ_2 value of 46 GM was measured at the concentration of 1.0×10^{-2} M. A 5–6 folds increase of the σ_2 value to 275 GM was achieved by simply decreasing the concentration to 1.0×10^{-3} M. The phenomena were much more pronounced for the dyad C_{60}(>DPAF-C_9) when the irradiation wavelength of 780 nm matched with two-photon absorption of DPAF-C_9 antenna. In this case, a 70-fold increase in the σ_2 value to 2190 GM was measured at 1.0×10^{-4} M, as compared with a 30 GM value at 1.0×10^{-2} M.[2] Since the 2PA measurement was performed at the focal area of the laser pulse, the signal intensity could be as low at 10^{-4} M leading to less accuracy in the calculation and line-fitting of the Z-scan data. Therefore, we used a medium concentration of 2.0–5.0×10^{-3} M for this study.

Figure S2. Open-aperture *Z*-scan profiles of (**a**) C_{60}(>DPAF-C_9)(>CPAF-C_{2M}) using laser pulses working at 780 nm with a 150-fs duration ([*C*] = 1.6 × 10^{-3} M in toluene), (**b**) C_{60}(>DPAF-C_{18})(>CPAF-C_{2M}) **3** and C_{60}(>DPAF-C_{18})(>CPAF-C_{2M})$_2$ **4** using 125 fs laser pulses operated at 800 nm and the input intensity of 270 GW/cm^2, and (**c**) fullerene derivatives C_{60}(>DPAF-C_9), C_{60}(>CPAF-C_9), **3**, and **4** using 125-fs laser pulses operated at 980 nm and the input intensity of 104 GW/cm^2. Measurements of (**b**) and (**c**) were carried out at the concentration of 5 × 10^{-3} M in toluene.

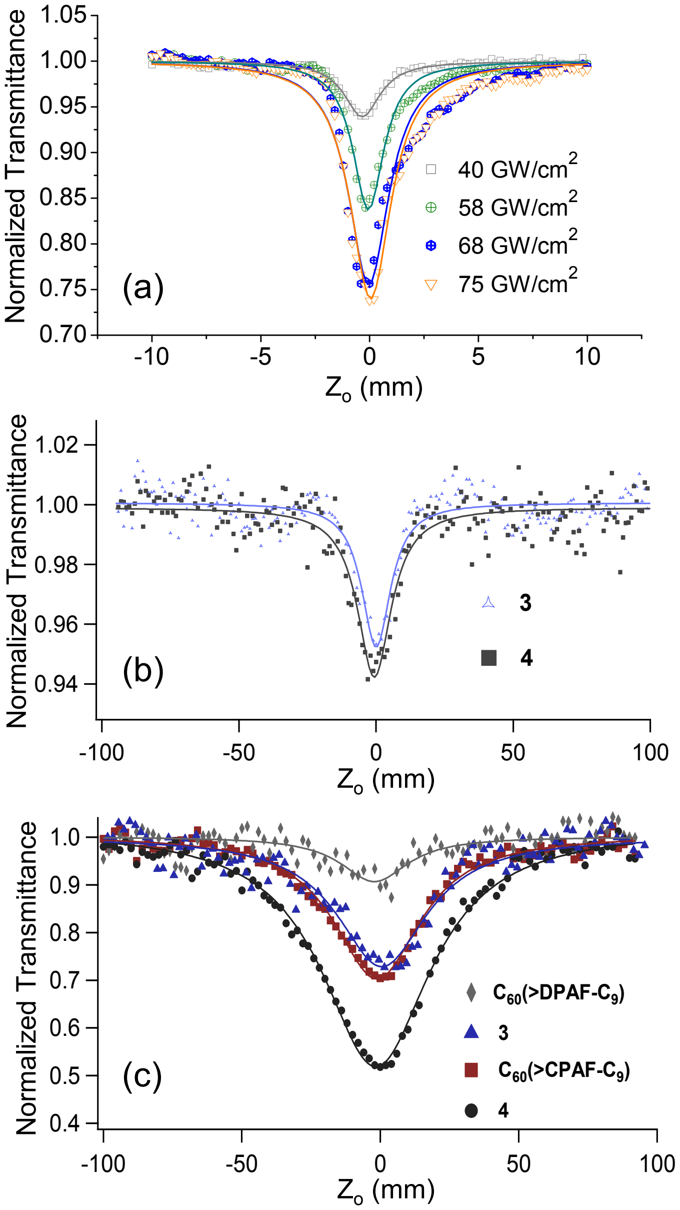

Table S1. Two-photon Absorption Cross-sections (σ_2) of Dyads C_{60}(>DPAF-C_9) and C_{60}(>CPAF-C_9), Hybrid Triads **3** and **3**-C_9, and the Hybrid Tetrad **4** at 780 nm [a].

Compound	[C]/M	*I*/GW cm^{-2}	*β*/cm GW^{-1}	σ_2/10^{-48} cm^4 s photon^{-1} molecule^{-1}
	1.0×10^{-4}	163	0.036	21.9 (2190 GM) [c]
C_{60}(>DPAF-C_9) [b]	1.9×10^{-3}	163	0.080	8.0 (800 GM) [c]
	1.0×10^{-2}	163	0.100	0.3 (30 GM) [c]
	1.0×10^{-3}	220	0.0065	2.75 (275 GM)
C_{60}(>CPAF-C_9) [d]	2.0×10^{-3}	220	0.0080	1.69 (169 GM)
	1.0×10^{-2}	220	0.0110	0.46 (46 GM)
**3**-C_9 [e]	1.6×10^{-3}	40	0.035	1.30 (130 GM)
**3**	5.0×10^{-3}	270	0.034	2.21 (221 GM)
**4**	5.0×10^{-3}	270	0.04	2.66 (266 GM)

[a] Measured using laser pulses working with a 125-fs duration and a repetition rate of 1.0 kHz, or with different conditions indicated. [b] Measured in CS_2 with 150-fs laser pulses at 800 nm. [c] Corrected value by deduction of the *β* value of CS_2 as the background correction. [d] Measured in THF with 226-fs laser pulses. [e] The triad C_{60}(>DPAF-C_9)(>CPAF-C_{2M}) measured in toluene with 150-fs laser pulses at 800 nm.

It is interesting to note that, even though the irradiation wavelength of 780 nm was not the best fit to the 2PA absorption λ_{max} of C_{60}(>CPAF-C_9) at 1000 nm, we still observed appreciable cross-section values. This could be owing to C_{60}-(antenna)$_x$ nanostructures in general acting as broadband NLO materials since the optical absorption of the chromophore component covers a broad wavelength range. In the comparison between hybrid triads **3**, C_{60}(>DPAF-C_{18})(>CPAF-C_{2M}), and **3**-C_9, C_{60}(>DPAF-C_9)(>CPAF-C_{2M}), we noticed that the use of long *n*-octadecyl chains (C_{18}) in **3** increased the solubility of nanostructure in toluene, as compared with that of the branched 3,5,5-trimethylhexyl (C_9) groups in **3**-C_9, and was able to produce a slightly higher 2PA σ_2 value of 221 GM, even though the measurement was made by a solution with 3 times higher in concentration. This value is much lower than that of C_{60}(>DPAF-C_9) in CS_2. Apparently, the solvent effect arising from CS_2 may play a significant role in the value of σ_2. For the hybrid tetrad **4**, the σ_2 value of 266 GM was slightly higher than that of **3** since the number of photoresponsive DPAF-C_{18} antenna was the same for both structures. An additional CPAF-C_{2M} antenna in **4** increased its $\sigma_2$value by 45 GM.

The second set of *Z*-scan experiments were performed in toluene using a pulse laser with a 125-fs pulse duration width at 980 nm to match with the linear absorption λ_{max} of the CPAF-C_{2M} antenna at 500 nm. As expected, the 2PA cross-section values of C_{60}(>DPAF-C_9) as 85 (I = 48) and 130 (I = 104 GM/cm^2) GM (Table S2) were found to be moderate at the concentration of 5.0×10^{-3} M since the 2PA energy at 980 nm is less than the main linear absorption λ_{max} (408 nm) of the DPAF-C_9 antenna. Higher 2PA σ_2 values of 450 (I = 48) and 538 (I = 104 GM/cm^2) GM for C_{60}(>CPAF-C_9) were measured in similar experiments, consistent with using well-matched absorption and excitation wavelengths. With an additional DPAF-C_{18} arm attached to the structure of **3**, no increase of σ_2 values was found with σ_2 = 504 GM (I = 104 GM/cm^2) in the same range as that of C_{60}(>CPAF-C_9). As the number of 980-nm-responsive antenna was increased to two in the structure of hybrid tetrad **4**, C_{60}(>DPAF-C_{18})(>CPAF-C_{2M})$_2$, the σ_2 values were doubled to 995 (I = 48) and 1100

(I = 104 GM/cm^2) GM. These data further demonstrated the suitability of **3** and **4** to be used as broadband NLO materials at 780–1000 nm.

Table S2. Two-photon Absorption Cross-sections (σ_2) of Hybrid Dyads C_{60}(>DPAF-C_9) and C_{60}(>CPAF-C_9), the Hybrid Triad **3**, and the Hybrid Tetrad **4** at 980 nm in Toluene [a].

Compound	[C]/M	I/GW cm^{-2}	β/cm GW^{-1}	σ_2/10^{-48} cm^4 s photon^{-1} molecule^{-1}
C_{60}(>DPAF-C_9)	5.0×10^{-3}	104	0.0198	1.3 (130 GM)
		48	0.0129	0.85 (85 GM)
C_{60}(>CPAF-C_9)	5.0×10^{-3}	104	0.0815	5.38 (538 GM)
		48	0.0677	4.5 (450 GM)
**3**	5.0×10^{-3}	104	0.0765	5.04 (504 GM)
		48	0.0468	3.1 (310 GM)
**4**	5.0×10^{-3}	104	0.166	11 (1100 GM)
		48	0.144	9.5 (950 GM)

[a] Measured using laser pulses working with a 125-fs pulse duration and the light intensity I indicated.

Nonlinear light-transmittance measurements of C_{60}(>CPAF-C_9), **3**, and **4** were performed as a function of irradiance intensity with 125-fs laser pulses at 980 nm with the concentration of 5×10^{-3} M in toluene. As shown in Figure S3(c), the *Z*-scans displayed positive signs for absorption nonlinearities with a decreasing trend of light-transmittance in the order of **4** > **3** ≈ C_{60}(>CPAF-C_9) > C_{60}(>DPAF-C_9) in solution with the input intensity of 104 GW/cm^2. Accordingly, the plot of transmittance (%) vs irradiance (GW/cm^2) of all samples showed a linear transmission (T = ~95%) with input intensity of up to 20 GW/cm^2. When the incident light intensity was increased above 30 GW/cm^2, the transmittance (%) began to deviate from the linear transmission line and decrease indicating the initiation of nonlinearity and the transmittance reduction effect. The transmitted fluence further departed from the linear line upon the increase in the incident fluence. A trend showing higher efficiency in reducing light-transmittance down to 50, 55, and 35% for C_{60}(>CPAF-C_9), **3**, and **4**, respectively, was observed with the increase of light intensity up to 110 GW/cm^2 (Figure S3). The observed improvement in lowering the transmitted light-intensity by **4** is consistent with its better nonlinear contribution that can be correlated to its higher fs 2PA cross-sections, as shown by the *Z*-scans.

Figure S3. Nonlinear transmittance of (**a**) C_{60}(>CPAF-C_9), (**b**) C_{60}(>DPAF-C_{18})(>CPAF-C_{2M}), and (**c**) C_{60}(>DPAF-C_{18})(>CPAF-C_{2M})$_2$ as a function of light intensity. All measurements were carried out with 125-fs laser pulses operated at 980 nm and the concentration of 5×10^{-3} M in toluene.

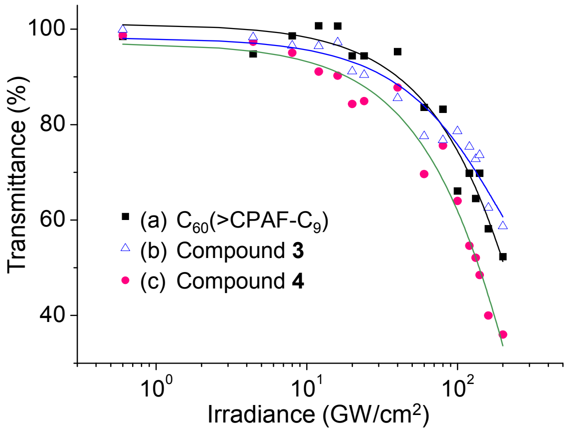

References

1. Sheik-Bahae, M.; Said, A.A.; Wei, T.H.; Hagan, D.J.; Van Stryland, E.W. *IEEE J. Quantum Electron.* **1990**, *26*, 760–769.
2. Elim, H.I.; Anandakathir, R.; Jakubiak, R.; Chiang, L.Y.; Ji, W.; Tan, L.-S. Large concentration-dependent nonlinear optical responses of starburst diphenylamino-fluorenocarbonyl methano[60]fullerene pentaads. *J. Mater. Chem.* **2007**, *17*, 1826–1838.
